# The activity of cell-free supernatant of *Lactobacillus crispatus* M247: a promising treatment against vaginal infections

**DOI:** 10.3389/fcimb.2025.1586442

**Published:** 2025-06-11

**Authors:** Giulia Santarelli, Roberto Rosato, Michela Cicchinelli, Federica Iavarone, Andrea Urbani, Maurizio Sanguinetti, Giovanni Delogu, Flavio De Maio

**Affiliations:** ^1^ Dipartimento di Scienze Biotecnologiche di Base, Cliniche Intensivologiche e Perioperatorie, Università Cattolica del Sacro Cuore, Rome, Italy; ^2^ Dipartimento di Scienze di Laboratorio ed Ematologiche, Fondazione Policlinico Universitario A. Gemelli IRCCS, Rome, Italy; ^3^ Mater Olbia Hospital, Olbia, Italy

**Keywords:** *Lactobacillus crispatus*, vaginal infections, probiotics, cell-free supernatant, LcM247

## Abstract

*Lactobacillus crispatus* is renowned for its antimicrobial properties, and some strains are used to treat vaginal dysbiosis, although the mechanisms underlying the antimicrobial properties remain elusive. We isolated *L. crispatus* M247 (LcM247) from a commercially available probiotic product Crispact^®^ and tested its antimicrobial activity against selected pathobionts such as *Escherichia coli*, *Klebsiella pneumoniae*, *Staphylococcus aureus*, *Streptococcus agalactiae*, *Enterococcus faecalis* and *Candida albicans* using both cocultures and testing the antimicrobial activity of cell-free supernatant (CFS) obtained from the culture of the probiotic strain. Furthermore, we demonstrate that CFS antimicrobial activity is pH dependent and that it is not affected by temperature and proteinase K treatment. Proteomic analysis suggests that this activity is mediated by S-layer secreted proteins. In a series of *in vitro* infection models, we infected Henrietta Lacks’ cervical eukaryotic cancer cells (HeLa) with *E. coli*, *S. agalactiae* and *C. albicans* at specific multiplicities of infection (MOIs) before the administration of LcM247, CFS, gentamicin or fluconazole alone or in combination with LcM247/CFS. We observed a slight decrease in the microbial burden following LcM247 administration, while treatment with CFS significantly reduced microbial growth compared to control and antimicrobial compounds. These results highlight the antimicrobial properties of LcM247 and its CFS and the likely mechanism of action that contributes to the eradication of common pathobionts. We show that actively replicating LcM247 is less efficient than its CFS, so the oral administration of LcM247 may result in treatment failure. Finally, the use of CFS may result in an upswing of the host *Lactobacillus* strains and promote the engraftment of *Lactobacillus* probiotic treatments.

## Graphical Abstract

The figure presents a concise three-part model. Firstly, it illustrates the standard approaches commonly used to treat vaginal infections, such as the systemic (oral) or localized administration of antibiotics, antifungal and probiotics. In the subsequent section, the experimental findings panel provides a summary of the *in vitro* data obtained, which indicated that both live *Lactobacillus crispatus* M247 (LcM247) and its cell-free supernatant exhibit the capacity to reduce the viability of urogenital pathogens over 24 hours. Notably, the cell-free supernatant (CFS) was found to be more efficacious in causing a more rapid decline in viability. In conclusion, the proposed solution delineates a pioneering two-step therapeutic regime: initially, an intravaginal pre-treatment with post-biotic metabolites (i.e. CFS) should be administered to modify the mucosal environment and reduce pathogen adhesion; subsequent administration of live LcM247 cells would then re-establish and maintain a protective, *Lactobacillus*-dominated flora.

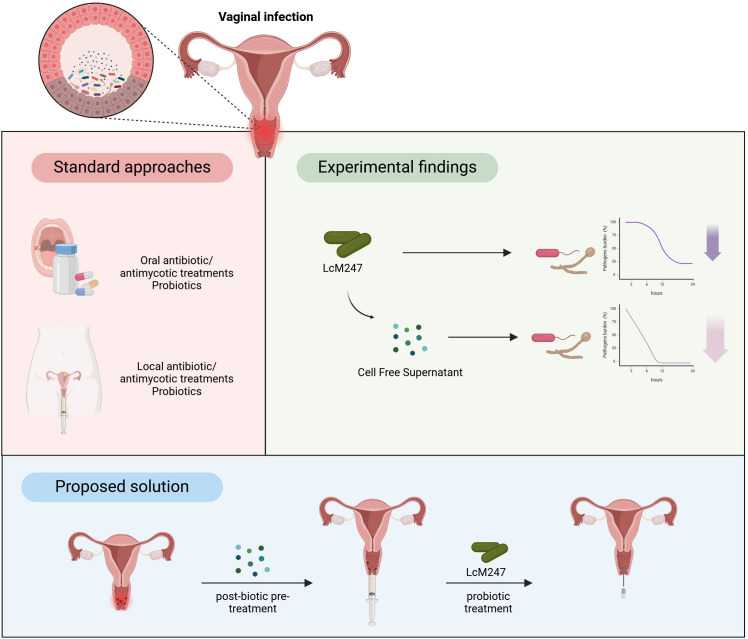

## Introduction

The vagina hosts a complex micro-ecosystem with billions of microorganisms that maintain a symbiotic and mutualistic relationship with the human host that preserve tissue homeostasis (Chen et al., 2021): the host provides a moist, rich in nutrients and warm habitat for several microbes that produce antimicrobial and anti-inflammatory compounds, which provide a first line of defence against harmful pathobionts (Joseph et al., 2021). This microbial balance can be disrupted by internal and/or external factors such as hormonal changes, age, immune system status, infections and antibiotic use, opening the possibility for opportunistic infections (Kumar et al., 2016; Shen et al., 2022). A classic example is bacterial vaginosis (BV), a condition marked by the disruption of the normal vaginal microbiota (Khedkar and Pajai, 2022), characterized by a decrease in lactobacilli and an overgrowth of anaerobic bacteria, including *Gardnerella vaginalis* and *Atopobium vaginae* (Abou Chacra et al., 2021; Abbe and Mitchell, 2023). BV potentially increases the risk of sexually transmitted infections (STIs) (van Houdt et al., 2018), pelvic inflammatory disease (PID) (Wiesenfeld et al., 2002; Ravel et al., 2021) and complications during pregnancy (Murphy and Mitchell, 2016; Feehily et al., 2020; Ravel et al., 2021; Masucci et al., 2023). On the contrary, vulvovaginal candidiasis (VVC), a yeast infection, is caused by an overgrowth of *Candida* species, particularly *Candida albicans* (Sobel, 2007; Nyirjesy et al., 2022), but unlike BV, it is not typically associated with an imbalance in the bacterial flora.

Under normal conditions, lactobacilli account for 70%–90% of the vaginal bacterial species associated with healthy premenopausal women (Hugenholtz et al., 2022), or they are positively linked to estrogen levels and contraceptive use and negatively linked to childbirth and breastfeeding (Lebeer et al., 2023); they are crucial to vaginal health because of their ability to produce antimicrobial molecules and lactic acid, which maintain the vaginal mucosa at an acidic pH (≈ 4.5) making the environment inhospitable for most microbes (O’Hanlon et al., 2013; France et al., 2020; Lebeer et al., 2023). However, the prevalence and abundance of *Lactobacillus* species in the vaginal microbiota are subject to a considerable interindividual variability (Greenbaum et al., 2019), although five distinct groups or cluster of vaginal microbial communities have been proposed and named as vaginal community state types (vCSTs) (Smith and Ravel, 2017; France et al., 2020). vCST I, II, III and V are characterized by the high abundance of *Lactobacillus crispatus*, *Lactobacillus gasseri*, *Lactobacillus iners* and *Lactobacillus jensenii*, respectively (Ma and Li, 2017), whereas vCST IV generally reflects a paucity of lactobacilli and an overgrowth of diverse anaerobes, typically associated with symptomatic BV (Norenhag et al., 2020; France et al., 2022). However, vCST IV does not always correspond to symptomatic BV; several studies, particularly in non-Western cohorts, have documented women harboring this diverse community but remain asymptomatic (Fettweis et al., 2014). Moreover, not all *Lactobacillus*-dominated CSTs confer the same health-promoting properties, with the vCST I showing a stronger association with vaginal healthy status compared to other vCST, suggesting that *L. crispatus* can be considered a microbial biomarker of a healthy vaginal microbiota (Lepargneur, 2016).

*L. crispatus* contributes to the vaginal environment by producing both the l-lactic and d-lactic acid isomers (Kaewsrichan et al., 2006; Borges et al., 2014) and synthesizing antimicrobial compounds, such as bacteriocins. Although their role in maintaining vaginal homeostasis remains under investigation, current evidence suggests that these antimicrobial peptides have the potential to regulate microbial communities in the vagina and inhibit the growth of competing microorganisms supporting the maintenance of a balanced microbiome (de Jong et al., 2006; Stoyancheva et al., 2014; Fontana et al., 2020). Furthermore, studies have still demonstrated that *L. crispatus* modulates the secretion of pro-inflammatory cytokines, which typically increase during bacterial vaginosis (Rose et al., 2012).

To date, the exogenous administration of *Lactobacillus*-based probiotics in treating BV to restore a healthy vaginal microbiome has attracted more attention from researchers (Cohen et al., 2020; Qi et al., 2023). *L. crispatus* M247 (LcM247) is a commercial probiotic that, following oral administration, colonizes the gut and vaginal environments, prompting the restoration of the physiological vaginal microbial equilibrium.

The aim of this study was to dissect the activity of LcM247 against various infectious agents, in different experimental *in vitro* models, using a set of potential vaginal pathobionts. Additionally, we studied the potential application of LcM247 supernatant as a natural antimicrobial agent.

## Materials and methods

### Bacterial cultures and growth conditions

The probiotic strain used in this study was isolated from the commercial product Crispact^®^ (Pharmextracta S.p.A., Piacenza, Italy), formulated in sachets. Each sachet contained not less than 20 billion colony-forming units (CFUs) of LcM247 (IDA: LMG-P-23257). Diluted powder of the sachet was spread on de Man, Rogosa and Sharpe (MRS) agar plates (Merck, Darmstadt, Germany). Colonies were inoculated in MRS broth and incubated for 48 hours at 37°C. Meanwhile, *Lactobacillus* were taxonomically identified at the species level using matrix-assisted laser desorption ionization–time of flight mass spectrometry (MALDI-TOF MS; Bruker, Kontich, Belgium) (Biswas and Rolain, 2013; Anderson et al., 2014).

The indicator strains were selected from our microbial bank or isolated from patients’ samples in the Department of Microbiology and Virology of IRCSS Fondazione Policlinico Agostino Gemelli, Rome (Italy). The pathogens *Escherichia coli* (ATCC 25922), *Klebsiella pneumoniae* (clinical isolate from urine), *Staphylococcus aureus* (ATCC 29213), *Enterococcus faecalis* (ATCC 29212), *Streptococcus agalactiae* (clinical isolate from vaginal swab) and *C. albicans* (ATCC 24433) were used as target microorganisms for the determination of antagonistic activity and cultured in Brain Heart Infusion (BHI) broth (Sigma-Aldrich, St. Louis, MO, USA) for the bacterial strain and in Sabouraud Dextrose broth (Sigma-Aldrich) for *C. albicans* at 37°C.

Each bacterial culture was mixed with pure sterile glycerol to reach a final concentration of 20% (Sigma-Aldrich), gently mixed and stored at −80°C.

To obtain cell-free supernatant (CFS), LcM247 colonies were inoculated in MRS broth and incubated for 48 hours at 37°C (reaching ~5 × 10^7^ CFU/mL) before centrifuging at 4,000 rpm for 10 min; CFS was harvested, filtered using 0.22-µm cellulose acetate membranes and stored at −80°C until use. An aliquot of CFS was plated onto MRS agar and cultured as previously described to assess that it was free of remaining bacteria.

### Assessment of the antimicrobial activity of LcM247 and its CFS

The antagonist effect of LcM247 against the selected pathogens was assessed in coculture assays and compared with the antimicrobial activity of its CFS and with the growth ability of each pathogen in MRS broth. The antagonism experiment was performed in a sterile 96-well plate (Corning^®^ Incorporated Life Sciences, NY, Corning, USA), which was mixed with 100 µL of indicator strains at 5 × 10^5^ CFU/mL [it is widely used as the starting concentration for many antimicrobial assays, including those presented in previously published papers (Rosato et al., 2024)] with 100 µL of sterile MRS broth or 100 µL of LcM247 at 5 × 10^7^ CFU/mL [it is the range of LcM247 CFU/mL reached in a 48-hour incubation to obtain CFS and according to (Rajab et al., 2020)] or 100 µL of CFS. The plate was incubated at 37°C under aerobic conditions and analyzed at 4 and 24 hours. The viable microbial cell counts (CFU/mL) of the indicator strains were reported as log_10_ reduction of the total count of CFU/mL plating on appropriate media (BHI agar, MacConkey agar, Columbia CNA agar and Sabouraud Dextrose agar), confirming strain identification using MALDI-TOF MS (Bruker). The turbidity of the medium in each well was evaluated using a spectrophotometer (Cytation 5, BioTek, Winooski, VT, USA) at λ = 600 nm.

To assess reciprocal *Lactobacillus* antagonism, 5 × 10^5^ CFU/mL of *L. iners* (isolated from a clinical vaginal sample) and LcM247 were incubated with serial dilutions of LcM247 CFS and *L. iners* CFS, respectively. The evaluation of bacterial viability was measured 24 hours post-incubation by counting CFUs as previously described.

### Evaluation of the antimicrobial activity of LcM247 CFS modified

To investigate the CFS antimicrobial effect, the supernatant was alkalinized by adding different volumes of 1 M NaOH until four different conditions were achieved: unaltered CFS (pH 4.5), CFS at pH 5.5, CFS at pH 7 and CFS at pH 9. In addition, the supernatant was diluted in MRS broth to obtain four serial dilutions with the following CFS/MRS concentrations: 0.5, 0.25, 0.12 and 0.06. The pH of each dilution was determined using pH indicator paper (pH range 1.0–10.0) (Sigma-Aldrich, USA). 100ul of the indicator strains with an initial concentration of 5 × 10^5^ CFU/mL were incubated with 100 µL of the above-mentioned conditions for 24 hours at 37°C in a 96-well plate with round-bottom wells. Once incubation was completed, bacterial replication or inhibition was assessed by observing the strain deposited at the bottom of the well as a dark button, and CFUs were determined after serial dilutions of the same wells.

### Whole-genome sequencing

*L. crispatus* M247 was grown onto MRS solid medium from frozen stock before being sub-cultured in MRS liquid medium for 48 hours at 37°C (reaching ~5 × 10^7^ CFU/mL) prior to DNA extraction. Highly pure genomic DNA was obtained using DANAGENE Microbial DNA according to the manufacturer’s instructions. A genomic library was prepared using the Illumina DNA Prep Kit (Illumina) and Nextera™ DNA CD Indexes (Illumina). Sequencing was performed using a MiSeq platform (Illumina), generating 250-bp read lengths. FastQ sequences were analyzed using CLC Genomic Workbench v24.0.2 (Qiagen, Valencia, CA, USA) (Nair et al., 2011; Sichtig et al., 2019). Briefly, raw data were quality-checked and then trimmed (using *QC for sequencing reads* and *Trim reads 3.0* plug-in with default parameters, respectively). Trimmed reads were additionally investigated for contamination using *Find Best Matches using K-mer Spectra* plug-in before *de novo* assembly using default parameters (minimum contig length of 200 bp). A total of 2.7 million paired-end reads were obtained, while ~2.2 million reads (average of 212 bp) were correctly assembled, obtaining a total of 1,202 contigs (199 > 1 kb, N50 = 11,896, GC 37.5%). Reads mapping to contigs (99.8% to exclude contaminations) showed a mean coverage of 250×, leading to ~2.0-Mb genome length with 100% of the reference genome covered (completeness). A total of 1,064 Coding DNA sequences (CDSs) (69.7%) were annotated with a DIAMOND hit of 1,526 CDS early detections. Genes encoding bacteriocins were detected by submitting the assembly fasta file to the BAGEL4 online software (de Jong et al., 2006; van Heel et al., 2018). Sequencing raw data have been deposited in the National Center for Biotechnology information (NCBI) Sequence Read Archive (BioProject accession number: PRJNA1222580, https://www.ncbi.nlm.nih.gov/bioproject/?term=PRJNA1222580).

### Enzymatic digestion and mass spectrometry analysis

Protein digestion was performed according to the filter-aided sample preparation (FASP) protocol that combines both purification and digestion (Wisniewski et al., 2009; Distler et al., 2016) of the proteins present in the LcM247 CFS, using MRS broth as a negative control. Briefly, 50 µg of proteins from each sample was reduced (Dithiothreitol (DTT) 8 mM in urea buffer − 8 M urea and 100 mM Tris), alkylated (Indole-3-Acetic Acid (IAA) 50 mM in urea buffer − 8 M urea and 100 mM Tris) and digested by trypsin on filter tubes Microcon^®^ Centrifugal Filter Devices (Merck Millipore Ltd., Cork, Ireland) at a final concentration of 1 μg/μL. Bottom-up proteomic analysis was performed using UltiMate™ 3000RSLCnano–HPLC System (Thermo Fisher Scientific, Waltham, MA, USA) coupled to a high-resolution Orbitrap Fusion Lumos Tribrid Mass Spectrometer (Thermo Fisher Scientific) with an Electrospray Ionozation (ESI) source. Peptides were separated via an PepMap RSLC C18 column 2 µM, 100 Å, 50 µm × 15 cm (Thermo Fisher Scientific) in gradient elution using an aqueous solution of Formic Acid (FA) (0.1%, v/v) as eluent A and Acetonitrile (ACN)/water (80:20, v/v) with 0.1% (v/v) FA as eluent B. The following step gradient was applied (run time 155 min): 3% eluent B and 97% eluent A (0–110 min), 20% eluent B and 80% eluent A (110–120 min), 40% eluent B and 60% eluent A (120–125 min), 90% eluent B and 10% eluent A (125–145 min) and 3% eluent B and 97% eluent A (145–155 min) (% values, v/v) at a flow rate of 0.300 μL/min. The injection volume was 5 μL (1 μg of peptides), with ion source type Nanospray Ionization (NSI), polarity positive (voltage 1,800 V) and ion transfer tube temperature of 275°C. The following MS parameters were set: the acquisition of high-resolution MS/MS spectra was carried out in data-dependent scan (DDS) mode using Orbitrap as detector, with a resolution of 120,000 in an m/z range of acquisition of 375–1,500– and higher-energy collisional dissociation (HCD) fragmentation. Samples were analyzed in analytical triplicate.

### Mass spectrometry data analysis

The bottom-up MS/MS data were elaborated using Proteome Discoverer 2.4.1.15 (Thermo Fisher Scientific) based on the SEQUEST HT algorithm (University of Washington, USA, licensed to Thermo Electron Corp., San Jose, CA, USA) against UniProt databases representing *Lactobacillus* (https://www.uniprot.org/taxonomy/1578) and *L. crispatus* (https://www.uniprot.org/uniprotkb/D5H222/entry) proteome. The setting parameters were as follows: minimum precursor mass 350 Da, maximum precursor mass 5,000 Da, total intensity threshold 0.0, minimum peak count 1, signal-to-noise (S/N) threshold 1.5, precursor mass tolerance 10 ppm, fragment mass tolerance 0.02 Da, use average precursor mass False, use average fragment mass False, maximum missed cleavage 2, minimum peptide length 6, maximum peptide length 144, Oxidation/+15.995 Da (M) as dynamic modification, Carbamidomethyl/+57.021 Da (C) as static modification and False Discovery Rate (FDR) at 0.01 (Strict) and 0.05 (Relaxed).

### Evaluation of cytotoxicity on eukaryotic cells

To measure cell viability under CFS treatment, Henrietta Lacks’ cervical eukaryotic cancer cells (HeLa) (ATCC^®^ CCL-2.2 TM) were cultured as described above and then plated at a final concentration of 5 × 10^5^ cells/mL in a 96-well plate. The cells were incubated overnight at standard atmosphere conditions (37°C and 5% CO_2_) until they were incubated with serial dilutions of CFS (CFS, CFS/2, CFS/4, CFS/8 and CFS/16). Untreated cells were used as a negative control, while cells treated with 2% Triton X-100 were used as a positive control. Triton X-100 interacts with lipid bilayers in a non-specific way, solubilizing bio-membranes and causing cell death (Koley and Bard, 2010; Mattei et al., 2017). Treated cells were incubated overnight at standard atmosphere conditions until the MTS Cell Proliferation Assay was performed to evaluate the cellular metabolic activity. Briefly, MTS reagent was added to each well and incubated for 4 hours at 37°C since absorbance was measured at 490 nm. Meanwhile, the cells were also fixed with Paraformaldehyde (PFA) 4% for 30 min and stained with crystal violet for 15 min to assess the monolayer integrity.

### Epithelial cell culture and infection

HeLa cells were cultured in Dulbecco’s modified Eagle’s medium (DMEM) (Euroclone, Pero, Italy) supplemented with 10% inactivated foetal bovine serum (FBS) (Euroclone, Italy), 1% l-glutamine (2 mM) (Euroclone) and 1% streptomycin–penicillin (100 µg/mL and 100 U/mL, respectively) (Euroclone) and were incubated at 37°C and 5% CO_2_. Adherent cells were washed with sterile warm Dulbecco’s Phosphate-Buffered Saline (DPBS) (Euroclone) and removed for experiments using 1× trypsin (5 μg/mL) in DPBS (Euroclone). Cells were counted and resuspended in DMEM supplemented with 2% Fetal Calf Serum (FCS) and 1% l-glutamine. Finally, cells were seeded in sterile 48-well plates (Euroclone) at a concentration of 5 × 10^5^ cells/mL, filling 0.5 mL/well, and incubated overnight until infection or treatment. HeLa cells were infected with *E. coli*, *S. agalactiae* and *C. albicans*, with a multiplicity of infection (MOI) of 100:1 and a MOI of 10:1 for yeast infection, and resuspended in the cell culture medium. Two hours post-infection, infected cells were washed three times with sterile warm DPBS to remove non-adherent bacteria and treated with *L. crispatus* (MOI 500:1, e.g. ~2.5 × 10^8^ CFU/mL), CFS (v/v 1:1 in cell medium), gentamicin or fluconazole at MIC and with the combination of antibiotic/antimycotic and LcM247 or CFS. Cells were incubated in standard atmosphere conditions for 24 hours, while CFUs were assessed by harvesting cell monolayers with 0.1 mL of sterile 0.01% Triton X-100 (Sigma-Aldrich, USA) 4 and 24 hours post-infection. Serial dilutions were carried out before plating on selective solid medium. Plates were then incubated at 37°C for 1 day. In the second experimental setting, cells were infected with *C. albicans* with a MOI of 10:1. Two hours post-infection, infected cells were washed thrice with sterile warm DPBS to remove non-adherent fungi. Subsequently, the cells were treated with fluconazole at the Minimum Inhibitory Concentration (MIC), CFS at v/v of 1:1 or fresh medium. Four hours later, treatments were removed, and cells were washed again to administer all conditions with *L. crispatus* (MOI 500:1). CFUs were assessed by harvesting cell monolayers with 0.1 mL of sterile 0.01% Triton X-100 at 24 and 48 hours post-infection. Serial dilutions were carried out before plating on selective solid medium. Plates were then incubated at 37°C for 1 day. Concurrently, to investigate LcM247 adherence on cell monolayers, cells were subjected to lysis 48 hours post-infection and plated on MRS agar. The plates were incubated at 37°C for 2 days before CFU counting.

### *In vivo* safety and effectiveness study on *Galleria mellonella* larva model

To assess the safety of the administered treatments (LcM247, CFS/2, gentamicin, fluconazole, gentamicin–LcM247, gentamicin–CFS/2, fluconazole–LcM247 and fluconazole–CFS/2) also in an *in vivo* model, healthy *G. mellonella* larvae measuring 2 to 2.5 cm in length and 0.3 to 0.45 g in body weight and showing no signs of melanization were selected for the experiment. Sterile water and Luria-Bertani (LB) broth (Sigma-Aldrich) were used as negative controls. Ten larvae were used to characterize each group and placed in a separate Petri dish. The larvae’s final left abdomen proleg was injected with 10 µL of each treatment, using 0.5-mL syringes with no dead volume, after sterilizing the region with 70% ethanol. Then, larvae were placed in an incubator at 37°C in the dark until the end of the experiment. The number of dead *G. mellonella* was scored every 24 hours for 4 days. Larvae were considered dead when they did not respond to touch. Survival curves were plotted using the Kaplan–Meier method, and differences in survival were calculated using the log-rank test (GraphPad Prism 10). Experiments were performed at least twice.

To evaluate the efficacy of the previously mentioned administered compounds, the same experimental setting was used to treat the *in vivo* infection model of *G. mellonella* larvae. Each larva’s final left abdomen proleg was injected with 10 µL of *E. coli* (5 × 10^5^ CFU/mL, that is, 5 × 10^3^ CFU/larvae), *S. agalactiae* (2 × 10^8^ CFU/mL, that is, 2 × 10^6–^ CFU/larvae) and *C. albicans* (5 × 10^7^ CFU/mL, that is, 5 × 10^5–^ CFU/larvae) using 0.5-mL syringes with no dead volume. Prior to treatment, 70% ethanol was used to sterilize the region. After 2 hours of 37°C incubation, 10-µL treatments were administered in the larvae’s final hind limb to investigate the efficacy against the infection. The larvae’s final right abdominal proleg was injected with 10 µL of each treatment. Sterile water was used as negative controls. Ten larvae were used to characterize each group and were separated into different Petri dishes. Infected and treated *G. mellonella* larvae were kept at 37°C in the dark. The number of dead *G. mellonella* was scored every 24 hours for 4 days. Larvae were considered dead when they did not respond to touch (**Figure 7A**). Survival curves were plotted using the Kaplan–Meier method, and differences in survival were calculated using the log-rank test of two nested experiments (GraphPad Prism 10).

### Statistical analysis

All data were generated from independent experiments with at least three technical replicates. Statistical significance was indicated using asterisks, with the following thresholds: *p* < 0.05 (*), *p* < 0.01 (**), *p* < 0.001 (***) and *p* < 0.0001 (****). Microsoft Excel (2024) and Graphpad Prism software v. 10 (GraphPad software) were used to collect and analyze the data. Data were expressed on a representative graph as mean ± SD and analyzed by one-way or two-way ANOVA comparison tests.

## Results

### *L. crispatus* M247 and its CFS inhibit the growth of indicator strains

When bacterial vaginosis occurs, including a consequence of antibiotic treatments (Chen et al., 2021; Abbe and Mitchell, 2023; Pendharkar et al., 2023), *L. crispatus*-based probiotics, including the strain M247 (LcM247) from Crispact^®^ (PharmExtracta S.p.A, Pontenure, Italy), are the most widely used commercially available probiotics to restore healthy microbiota, although their activity is not fully understood (Cesena et al., 2001; Di Pierro et al., 2019).

**Figure 1:**
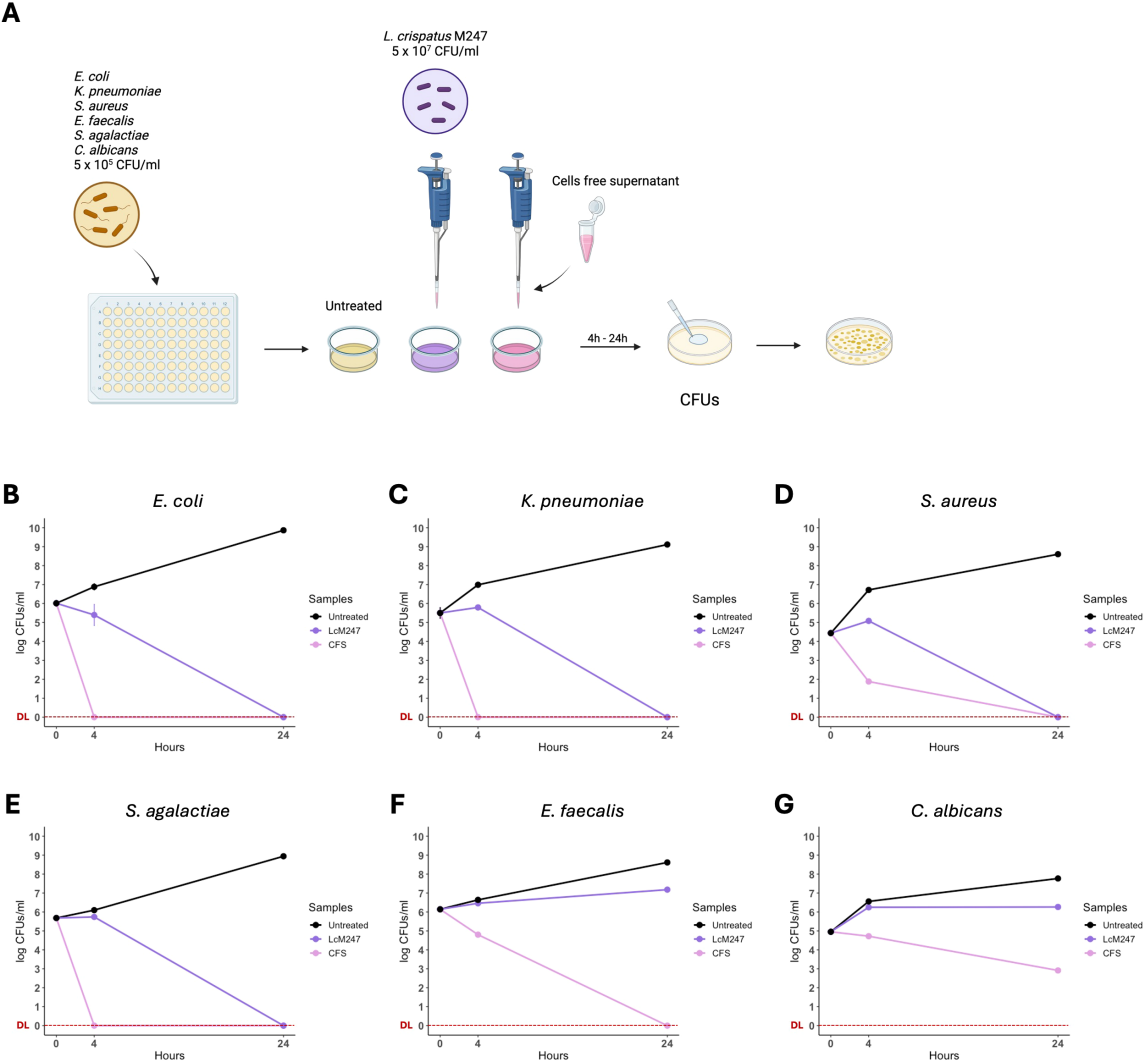
*Lactobacillus crispatus* M247 and its cell-free supernatant (CFS) differently reduced microbial burden in the short-term period. Antimicrobial properties of *L. crispatus* M247 (LcM247) and its CFS were assayed by incubating a suspension of 5 × 10^5^ CFU/mL of *Escherichia coli*, *Klebsiella pneumoniae*, *Staphylococcus aureus*, *Streptococcus agalactiae*, *Enterococcus faecalis* and *Candida albicans*, named as indicator strains. LcM247 was used at the final concentration of 5 × 10^7^ CFU/mL, while CFS was added with a v/v ratio of 1:1 with the microbial cultures **(A)**. Colony-forming units (CFUs) were measured at 4 and 24 hours post-treatment and are presented as line plot reporting the mean ± SD in log10 scale of three repeated experiments **(B–G)**.

The LcM247 antagonist effect and its CFS were assessed against six main microbial indicator species: *E. coli*, *K. pneumoniae*, *S. aureus*, *E. faecalis*, *S. agalactiae* and *C. albicans*. Each indicator strain was incubated with LcM247 at a ratio of 1:100 (CFUs/CFUs) or with LcM247 CFS (1:1 v/v). CFUs were assessed at 4 and 24 hours post-incubation. A schematic representation of the experimental setting is reported in **Figure 1A**.

A marked inhibitory effect was only observed against *E. coli*, in the presence of LcM247, accounting for a reduction by 10% compared to the initial infection solution (*p* < 0.01) (**Figure 1B**). Conversely, for the other indicator strains, LcM247 co-treatment was not able to prevent bacterial replication (**Figures 1C–F**). Finally, no fungistatic effect was still observed on *C. albicans* replication at 4 hours (**Figure 1G**).

A significant activity was observed following 24 hours of incubation with LcM247. Indeed, LcM247 exerted a robust bactericidal activity after 24 hours on selected bacteria, leading to the complete elimination of *E. coli*, *K. pneumoniae*, *S. aureus* and *S. agalactiae* (*p* < 0.0001) (**Figures 1B–E**). Conversely, the activity of LcM247 against *E. faecalis* and *C. albicans* was negligible in comparison with that of the infection solution at 4 hours post-incubation (**Figures 1F, G**).

To investigate whether, similar to what is known for lactobacilli, the antimicrobial activity of LcM247 is associated with secreted molecules, the LcM247 CFS was collected, and the microbial indicators chosen with this preparation were incubated. Interestingly, 4-hour incubation with CFS was sufficient to completely inhibit the growth of *E. coli*, *K. pneumoniae* and *S. agalactiae* (*p* < 0.0001) (**Figures 1B, C, E**), with a 5-log CFU reduction of *S. aureus* and a 2-log CFU reduction for both *E. faecalis* and *C. albicans* CFUs, compared to untreated controls (**Figures 1D, F, G**). Notably, no detectable colonies were found after 24 hours of incubation of CFS with all the analyzed bacterial strains (*p* < 0.0001) (**Figures 1B–F**), whereas incubation with *C. albicans* resulted in a 5-log CFU reduction compared to the untreated control (*p* < 0.0001) (**Figure 1G**). Our results demonstrate the antimicrobial activity of LcM247, which is conserved in its CFS.

Strengthening these hypotheses, **Supplementary Figure S2** illustrates the significant, dose-dependent antimicrobial effect of LcM247 CFS against *L. iners* bacterial cells. Notably, the reciprocal experiment demonstrated that *L. iners* CFS failed to exert any detectable inhibitory effect on LcM247, whose growth remained stable across all conditions tested. These results suggest that *L. crispatus* secretes antimicrobial compounds absent in *L. iners* supernatant, prompting us to further investigate the bioactive content of LcM247 CFS.

### LcM247 CFS antimicrobial activity is pH dependent

Numerous studies have demonstrated that the CFS produced by lactobacilli plays a crucial role in antimicrobial activity, primarily due to its content of lactic acid, which drives the decrease in the environmental pH, and bacteriocins, peptide-based toxins, target bacterial or fungal strains by disrupting their membranes (Abdul-Rahim et al., 2021; Mani-Lopez et al., 2022). Hence, we investigated the antimicrobial effect of CFS to elucidate whether this was concentration- or pH-dependent.

**Figure 2:**
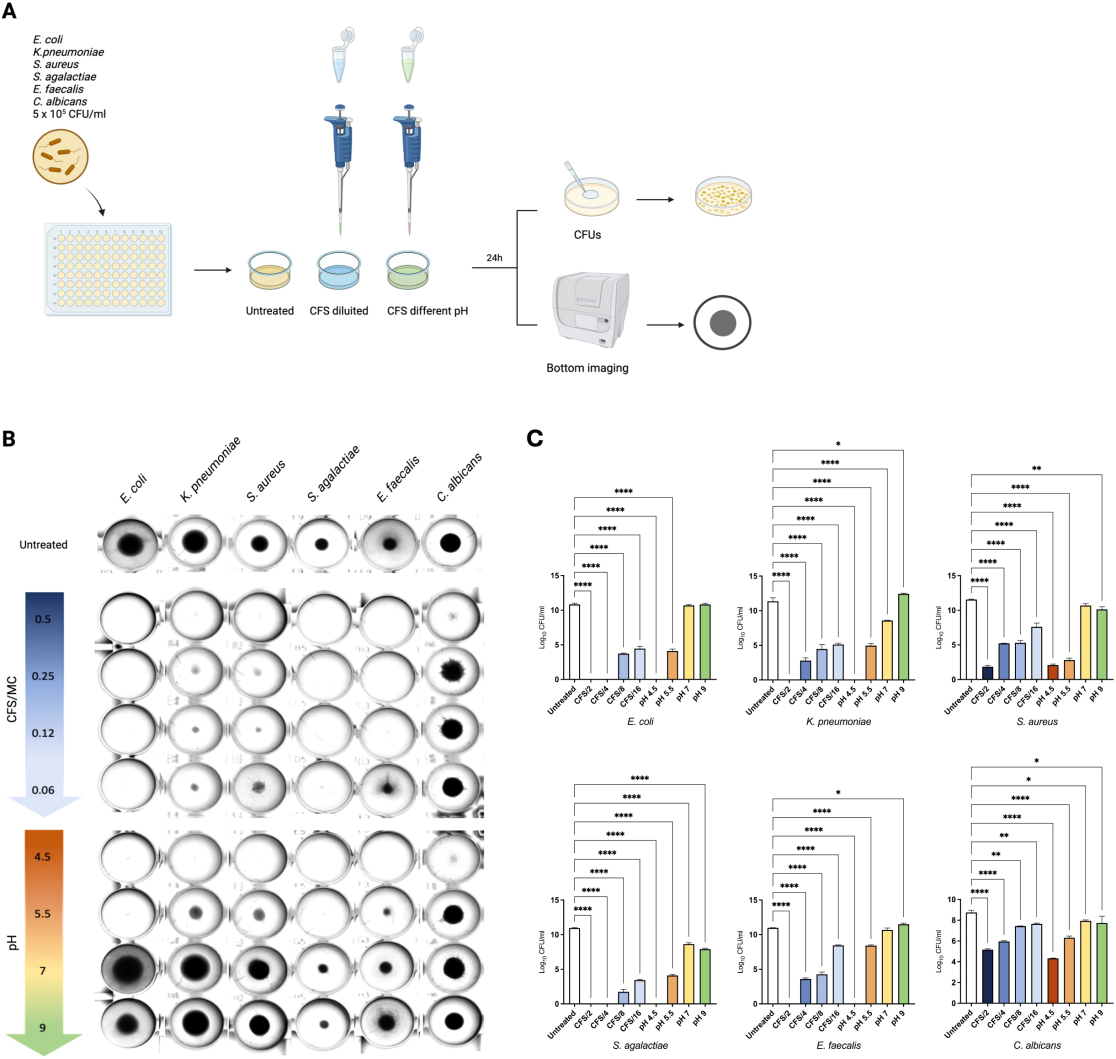
Acidic pH plays a key role in mediating LcM247 CFS antimicrobial activity. CFS antimicrobial activity was assayed using serially diluted CFS and alkalinized CFS by adding different volumes of 1 M NaOH. CFS was diluted in MRS broth to achieve final CFS/MRS v/v ratio of 1:1, 1:2, 1:4 and 1:8 (i.e. CFS/2, CFS/4, CFS/8 and CFS/16, respectively) (as reported in **Supplementary Figure S1**, pH was maintained at value of 4.5). CFS alkalinization generated unaltered CFS, which corresponded to CFS/2 (pH 4.5), CFS at pH 5.5, CFS at pH 7 and CFS at pH 9. Modified CFS solutions were incubated with a suspension of the indicator strains *Escherichia coli*, *Klebsiella pneumoniae*, *Staphylococcus aureus*, *Streptococcus agalactiae*, *Enterococcus faecalis* and *Candida albicans* at final concentration of 5 × 10^5^ CFU/mL for 24 hours at 37°C in a 96-well plate with round-bottom wells. Microbial growth in MRS broth was used as a control **(A)**. At the end of the incubation, the microbial growth was assessed by observing the strain pellet deposited at the bottom of the well using the Cytation instrument **(B)** and measuring colony-forming units (CFUs) determined after serial dilutions of the last well showing microbial growth **(C)**. Data from three repeated experiments are presented as bar plot reporting the mean ± SD on log_10_ scale. LcM247, *Lactobacillus crispatus* M247; CFS, cell-free supernatant; MRS, de Man, Rogosa and Sharpe. Statistical signifcance was indicated using asterisks, with the following thresholds: p < 0.05 (*), p < 0.01 (**) and p < 0.0001 (****).

Briefly, four dilutions of CFS, appropriately diluted in MRS medium (v/v CFS/MRS 1:1, 1:4, 1:8 and 1:16 named as CFS/2, CFS/4, CFS/8 and CFS/16, respectively), were obtained and tested against the previously mentioned microbial indicator strains. CFS was also alkalinized by adding different volumes of 1 M NaOH, creating four modified CFS with different pH values (pH 4.5, representing the untreated v/v CFS/MRS 1:1, pH 5.5, pH 7 and pH 9). All modified CFSs were incubated with the indicator strains for 24 hours at 37°C in a 96-well plate with round-bottom wells. At the end of the incubation, the growth was assessed by observing the strain pellet deposited at the bottom of the well and by counting CFUs for each condition (**Figure 2A**).

As shown in **Figure 2B**, the CFS/2, corresponding to CFS showing pH 4.5, significantly inhibited the microbial growth of all indicator strains, compared to the untreated specimens. However, the bacterial pellet at the bottom of the wells appears almost comparable for the other diluted CFSs (CFS/4, CFS/8 and CFS/16), while progressive alkalization was directly associated with microbial growth. Indeed, CFSs at pH 7 and pH 9 lose the ability to block bacterial and fungal replication. These results suggest that acidic pH is necessary to reduce microbial growth.

To corroborate this finding, we measured microbial growth by evaluating CFUs following treatment. As shown in **Figure 2C**, CFS/2 and CFS at pH 4.5 confirmed a complete growth inhibition of *E. coli*, *K. pneumoniae*, *S. agalactiae* and *E. faecalis* (*p* < 0.0001). Conversely, the same treatments did not result in the complete elimination of *S. aureus* and *C. albicans*, where the reduction accounted for 9.5 log and 4 log (*p* < 0.0001), respectively. Similarly, CFS/4 incubated with *E. coli* and with *S. agalactiae* led to complete bacterial elimination (*p* < 0.0001), while a reduced effect was observed for *K. pneumoniae*, *S. aureus* and *E. faecalis* (~8 log, 6 log and 7 log of reduction, respectively, *p* < 0.0001) and *C. albicans* (~2 log, *p* < 0.0001) compared to the untreated controls. CFS/8 and CFS/16 treatments showed a significant but incomplete antimicrobial effect, reducing all gram-negative bacteria and *S. agalactiae* burden by 9 log and 7.5 log (*p* < 0.0001), respectively, and *S. aureus* and *E. faecalis* by 7 log and 4 log (*p* < 0.0001), respectively, while reducing *C. albicans* growth by less than 1 log (*p* = 0.0024 for CFS/8 and *p* = 0.0078 for CFS/16). Importantly, the pH values of CFS/2, CFS/4, CFS/8 and CFS/16 were not affected by dilution in MRS liquid medium (**Supplementary Figure S1**). As previously observed, CFU measurement highlighted that CFS at pH 5.5 maintained a significant antimicrobial activity, reducing bacterial growth from a minimum of 2 log for *C. albicans* (*p* < 0.0001) to a maximum of 8 log for *S. aureus* (*p* < 0.0001). Importantly, minimal reduction in microbial load was evidenced when the CFS pH was increased to 7 and 9 (*p* = 0.954 and *p* > 0.999 for *E. coli*, *p* < 0.0001 and *p* = 0.062 for *K. pneumoniae*, *p* = 0.079 and *p* = 0.005 for *S. aureus*, *p* < 0.0001 and *p* < 0.0001 for *S. agalactiae*, *p* = 0.395 and *p* = 0.045 for *E. faecalis* and *p* = 0.395 and *p* = 0.045 for *C. albicans*). These results indicate that the LcM247 CFS antimicrobial activity is pH dependent.

### *Lactobacillus* CFS antimicrobial activity is not impaired by heat or proteinase treatment

**Figure 3:**
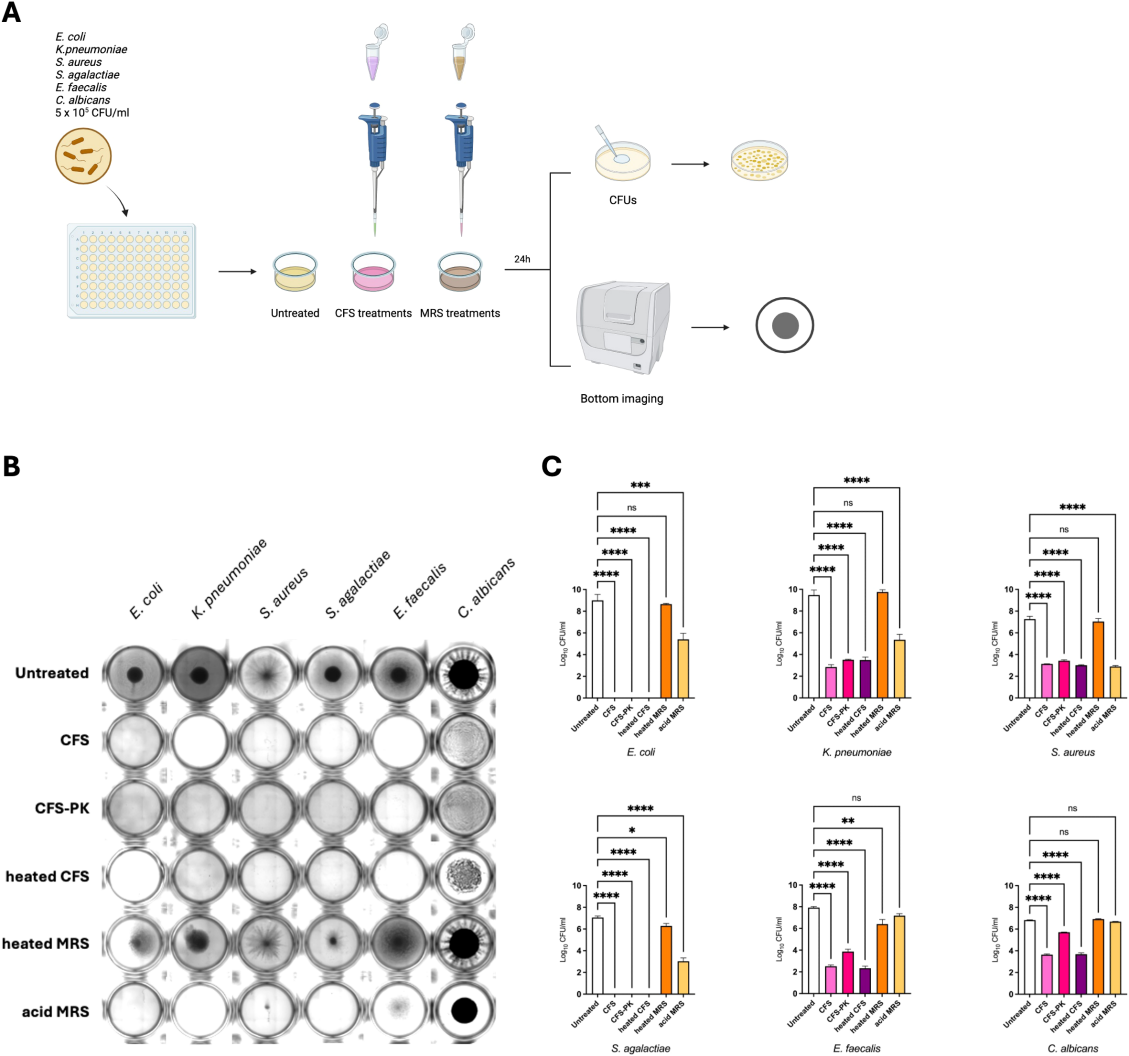
LcM247 CFS maintains antimicrobial activity following heat inactivation and enzymatic treatment. LcM247 CFS was heat inactivated (98°C) or enzymatically treated using proteinase (K) MRS broth previously heated at 98°C for 1 hour and acidified with HCl to reach pH value of 4.5 was used as negative control. Modified CFSs were prepared with 5 × 10^5^ CFU/mL of the indicator strains (*Escherichia coli*, *Klebsiella pneumoniae*, *Staphylococcus aureus*, *Streptococcus agalactiae*, *Enterococcus faecalis* and *Candida albicans*) with a v/v 1:1 ratio and incubated in standard atmosphere conditions for 24 hours **(A)**. At the end of the incubation, the microbial growth was assessed by observing the strain pellet deposited at the bottom of the well using citation instrument **(B)** and measuring colony-forming units (CFUs) determined after serial dilutions of the last well showing microbial growth **(C)**. Data from three repeated experiments are presented as bar plot reporting the mean ± SD on log_10_ scale. Statistical signifcance was indicated using asterisks, with the following thresholds: ns: not significant, p < 0.05 (*), p < 0.01 (**), p < 0.001 (***) and p < 0.0001 (****).

To elucidate the role of bacteriocins, the antimicrobial activity of LcM247 CFS was assessed through heat inactivation and proteinase K proteolytic enzymatic treatment. After exposure to 98°C for 1 hour and enzymatic treatment at 56°C for 30 min, undiluted CFS was tested against the indicator strains for 24 hours at 37°C before CFU evaluation (**Figure 3A**). Intriguingly, microbial growth was inhibited even when CFS was heated or enzymatically treated, showing bacterial or fungal pellets comparable to specimens treated with unmodified CFS. Importantly, heated MRS did not affect indicator strains’ growth, unlike acidified MRS, which reduces bacterial pellet size, sometimes making them macroscopically invisible. Conversely, *C. albicans* appeared full-grown in all conditions (**Figure 3B**). CFU analysis confirmed that the inhibitory effect of CFS significantly reduced the microbial load from a minimum of 3 log for *C. albicans* (*p* < 0.0001) to a maximum of 9 log for *E. coli* (*p* < 0.0001), resulting in a complete elimination of *E. coli* and *S. agalactiae* (100% reduction) (**Figure 3C**). These findings were comparable to those observed with heat-inactivated CFS, which showed the same inhibitory effect (*p* < 0.0001 for each indicator strain and a 100% reduction for *E. coli* and *S. agalactiae*). Similarly, proteinase K treatment did not significantly affect the CFS antimicrobial role. Indeed, despite this enzymatic treatment, the CFS still showed a significant ability to decrease microbial growth, which, although slightly less pronounced for *E. faecalis* and *C. albicans*, was comparable to the inhibitory effect of unmodified CFS (*p* < 0.0001). Importantly, the heated MRS had no antimicrobial activity on the indicator strains, which was comparable to that of the normal growth of untreated controls (*p* = 0.742 for *E. coli*, *p* = 0.851 for *K. pneumoniae*, *p* = 0.569 for *S. aureus*, *p* = 0.013 for *S. agalactiae*, *p* = 0.002 for *E. faecalis* and *p* = 0.491 for *C. albicans*). Interestingly, the acidified MRS reduced the microbial burden, especially for those bacteria that were observed to be most sensitive to the acidic microenvironment, in particular *E. coli*, *K. pneumoniae*, *S. aureus* and *S. agalactiae* (*p* < 0.0001). Conversely, more resistant strains, such as *E. faecalis* and *C. albicans*, did not show a reduction in CFUs after treatment with acidic MRS (*p* = 0.061 and *p* = 0.184, respectively). Taken together, these results suggest that CFS pH-dependent antimicrobial properties are mediated by heat- and protease-resistant microbial components.

### Genomic and proteomic analysis of LcM247

While it is well known that *Lactobacillus* species produce several metabolites (organic acids and hydrogen peroxide) that are important to maintain a favorable environment, little information is available on the antimicrobial activity of secreted proteins (i.e. bacteriocins). We performed a genomic analysis of the LcM247 genome and a proteomic analysis of its CFS to identify potentially synthesized and secreted peptides/proteins that may mediate the antimicrobial properties. The general genomic characteristics of LcM247 after *de novo* assembly are reported in the Materials and Methods section. Bacteriocin-encoding genes were predicted by uploading the assembly *fasta* file into BAGEL4, which is specifically designed for bacteriocin mining in prokaryotic genomes (van Heel et al., 2018). BAGEL4 analyzed the sequence and identified the genome/contig areas of interest (AOIs) that encode bacteriocin-like peptides (Li et al., 2022). The analysis revealed seven AOIs within the genome containing bacteriocin-associated genes (**Supplementary Table S1**). Putative bacteriocin genes, showing similarity to the bacteriocin genes encoding for *Enterolysin A* (match 67.299% and 59.615% in comparison with BAGEL4 reference amino acid sequence; for both, no bit score was reported) and *Penocin A* (match 34.783% with bit score 50.061 and match 36.765% with bit score 39.276 in respect to BAGEL 4 reference amino acid sequence) were detected twice in two separate regions within the LcM247 genome assembly. Of note, the reference strain (CP088015) showed only six AOIs, suggesting that *de novo* alignment based on the short-read sequencing may be impaired by technical errors and not exactly evidence of gene duplication.

The proteomic composition of the CFS was analyzed to identify secreted peptides that may be potentially responsible for its antimicrobial activity. In **Table 1**, we summarized the major features revealed in the LcM247 CFS, which were assessed by at least two peptides, which refers to an accurate protein identification (i.e. the detection of at least two unique peptides per protein). Contrary to what we anticipated, the proteomic analysis did not detect any bacteriocin in the *Lactobacillus* secretome (**Table 1**). Indeed, we could not detect any bacteriocin when searching for at least two peptides (**Supplementary Table S2**) in the *L. crispatus* database or the *Lactobacillus* genus database (**Supplementary Tables S3**, **S4**). Instead, we observed a predominance of surface-associated proteins, many of which appear to be involved in *Lactobacillus* fitness.

**Table 1:** Comprehensive proteomic profiling of secreted proteins in the CFS from LcM247.

Protein	Number of proteins	Accession number	Function
*S-layer protein*	6	V5ENE5 A0A125P6L8 A0A4R6CRA1 Q9Z4J9 A0A2N5L0N4 A0A4R6CVY3	Surface layer protein
*Signal peptide*, *YSIRK family*	3	A0A135ZB87 A0A135Z649 A0A4Q0LRA7	Transport of nascent proteins to the cross-wall site or extracellular
*Aggregation promoting factor*	2	K1MS77 Q5W5V4	Protein involved in the conjugation and auto-aggregation
*Thioredoxin*	2	K1P134 D5H1I1	Redox protein
*Arylsulfatase*	1	A0A135ZF41	Hydrolase protein
*Cell division protein*	1	A0A4Q0LUV9	Protein involved in cell division
*Choloylglycine hydrolase*	1	A0A109DF58	Protein involved in bacterial fitness
*Copper oxidase*	1	A0A135ZG11	Protein that oxidizes its substrate by accepting electrons at a mononuclear copper centre and transferring them to a trinuclear copper centre
*DJ-1 family protein*	1	K1PE20	Protein deglycase that repairs methylglyoxal- and glyoxal-glycated proteins
*Extracellular solute-binding protein*	1	A0A5M9Z202	Active transport of solute across the cytoplasmic membrane
*Glyceraldehyde-3-phosphate dehydrogenase*	1	K1N3X9	
*Iron-sulfur cluster biosynthesis*	1	Q5W5V7	Fe/S biogenesis protein
*Lipoprotein*	1	A0A6A1Z710	May interact with Toll-like receptor (TLR) 2 of the host innate immune system
*LPXTG cell wall anchor protein*	1	A0A135Z635	LPxTG motif in surface proteins of gram-positive bacteria recognized and cleaved by a conserved transpeptidase enzyme called sortase
*LysM domain-containing protein*	1	K1NFP0	Protein module involved in binding peptidoglycan in bacteria
*Membrane transport protein MMPL*	1	A0A109DDP8	Protein involved in transport of other proteins
*PDZ domain-containing protein*	1	A0A109DRM6	Signaling protein
*Phosphate-binding protein*	1	A0A6A1Z4S3	
*PTS system glucitol/sorbitol-specific IIa component*	1	V5EPR9	Phosphoenolpyruvate-dependent sugar phosphotransferase system (PTS) involved in carbohydrate transport
*Putative neprilysin*	1	A0A135YME3	Neutral endopeptidases
*Small ribosomal subunit protein*	1	D5GXZ3	
*UPF0342 protein HMPREF9249_00543*	1	K1P126	Protein involved in the formation of biofilm
*DUF1002 domain-containing protein*	1	A0A120DI73	Unknown function
*Uncharacterized protein*	2	V5EKL8 A0A4V3BIF2	Unknown function

The table displays the proteins identified in the CFS that were blasted with *Lactobacillus crispatus* database and confirmed by the presence of at least two peptides. The table also illustrates the number of proteins identified and provides the corresponding accession numbers, along with their known or predicted functions.

CFS, cell-free supernatant; LcM247, *L. crispatus* M247.

Of note, our analysis highlighted several proteins associated with the S-layer, which is critical for the ability of *Lactobacillus* to exert antimicrobial effects, or peptides belonging to proteins involved in aggregation-promoting factors and signal peptides of the YSIRK family, suggesting mechanisms related to bacterial adherence and signal transmission (Edelman et al., 2012).

Additionally, structural proteins and enzymes, which are involved in redox reactions and the overall bacterial fitness, were identified. Unfortunately, a label-free analysis measuring the peptide abundance was not possible due to the lack of a reliable *Lactobacillus* list in databases. Nevertheless, coverage and score measures suggested that S-layer peptides represented the most relevant CFS component. Conversely, the lack of bacteriocins in the CFS points toward a negligible role of these peptides in our experimental settings.

### Evaluation of the CFS antimicrobial effect during microbial infection of a cervical epithelial cell line

**Figure 4:**
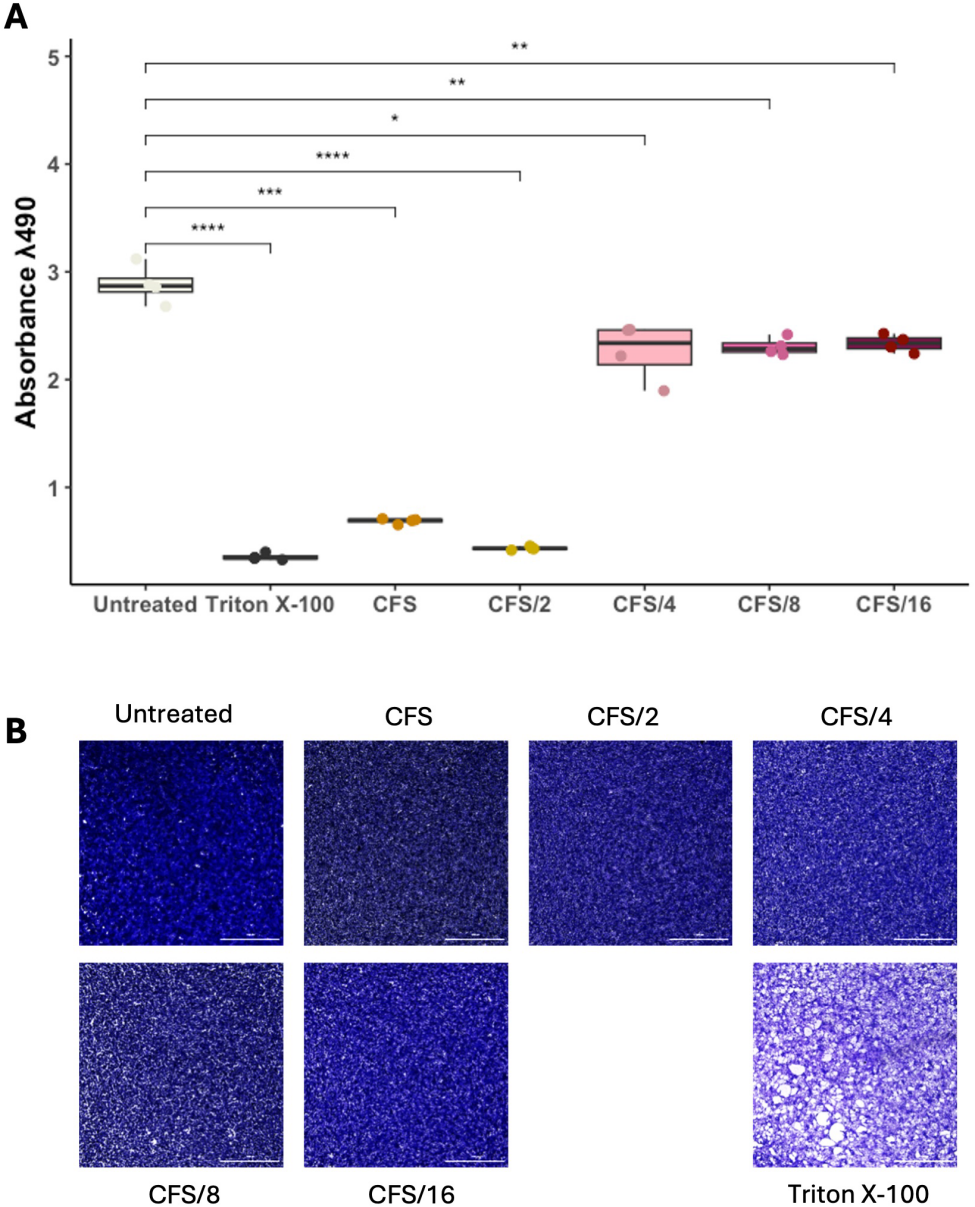
LcM247 CFS shows good biocompatibility on eukaryotic cells. HeLa cells were seeded in a 96-well plate and treated with serial dilutions of CFS (CFS, CFS/2, CFS/4, CFS/8 and CFS/16) when 90% cell monolayer was reached. Untreated cells and cells treated with 2% Triton X-100 were used as negative and positive controls, respectively. One day post-incubation, cells’ metabolic activity was evaluated using MTS assay **(A)**. Results of three repeated experiments were acquired using the Cytation instrument and are reported as box plot chart. Cellular monolayer was fixed and stained using crystal violet staining. Images were acquired using Cytation instrument and processed with ImageJ software to evaluate monolayer’s integrity **(B)**. LcM247, *Lactobacillus crispatus* M247; CFS, cell-free supernatant. Statistical signifcance was indicated using asterisks, with the following thresholds: p < 0.05 (*), p < 0.01 (**), p < 0.001 (***) and p < 0.0001 (****).

Our findings on axenic cultures have suggested a rapid and efficient activity of CFS compared to live LcM247, offering a novel potential therapeutic strategy. Hence, we first assessed CFS cytotoxicity on HeLa cells by measuring the metabolic activity and monolayer integrity using MTS and crystal violet assays, respectively (De Maio et al., 2021; Rosato et al., 2024; Santarelli et al., 2024). As reported in **Figure 4A**, CFS and CFS/2 appeared to impair cell viability, while cells treated with CFS/4, CFS/8 and CFS/16 showed values up to untreated cells. To corroborate these findings, cell monolayer integrity was assessed through staining with a crystal violet solution. This demonstrated that none of the CFS dilutions caused damage to HeLa cells (**Figure 4B**). The phenomenon under investigation can be attributed to the sensitivity of the MTS reagents to even slight pH variations or certain metabolites present in undiluted or slightly diluted CFS. These factors can lead to an artefact in the MTS signal.

**Figure 5:**
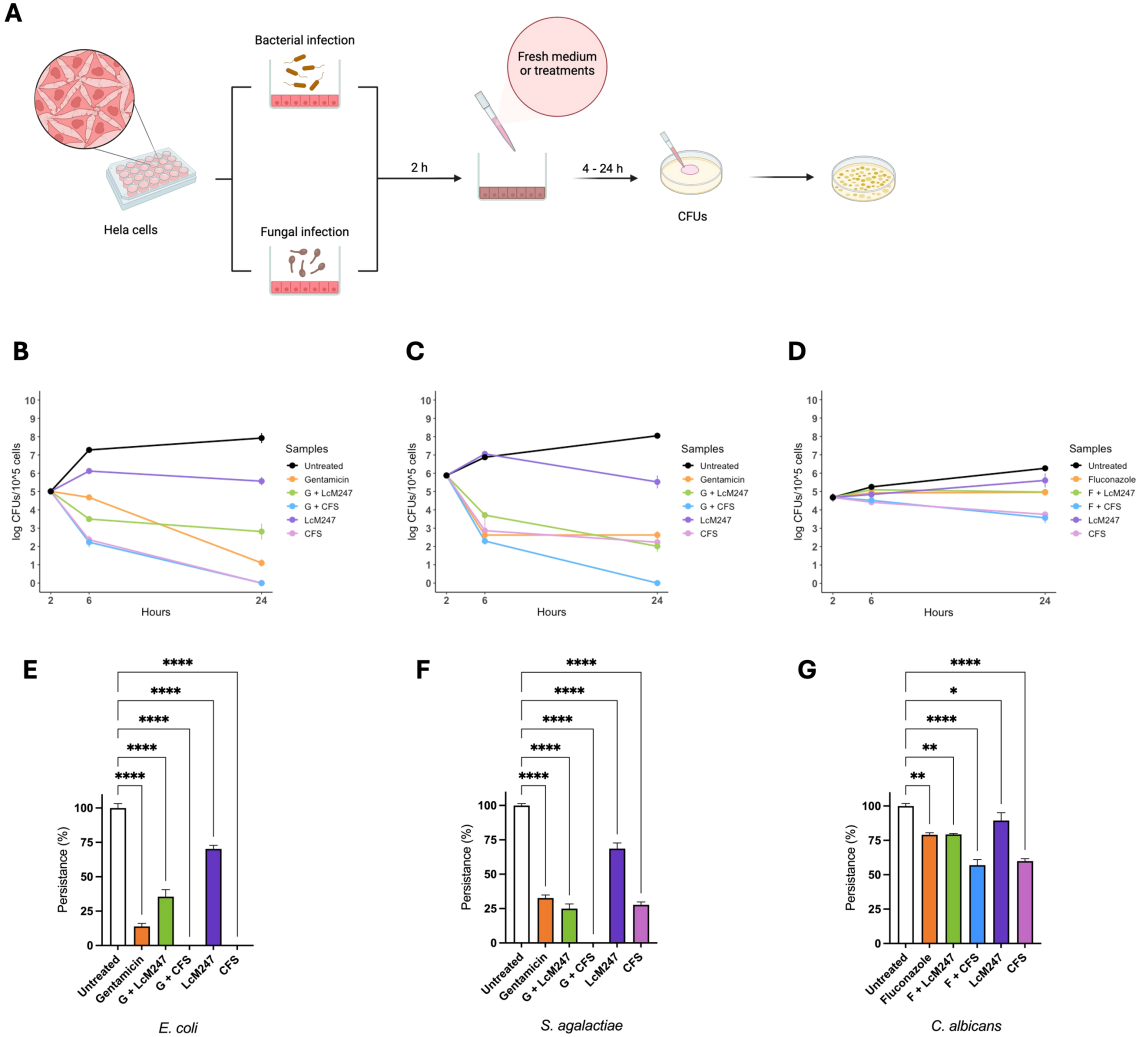
LcM247 CFS reduces microbial burden in cervical epithelial cells. HeLa cells were infected with selected indicator strains *Escherichia coli*, *Streptococcus agalactiae* and *Candida albicans* with multiplicity of infection (MOI) of 100:1, 100:1 and 10:1, respectively. Two hours after infection, cells were washed three times and treated with LcM247 (MOI = 500:1), CFS (CFS v/v 1:1 in culture medium), antibiotic (gentamicin) or antimycotic (fluconazole) at MIC and finally a combination of antibiotic/antimycotic with LcM247 or CFS **(A)**. Four- and twenty-four-hours post-incubation, colony-forming units were assessed. Line plot showing Log CFUs/10^5^ cells generated in three repeated experiments. Persistence at 24 hours was reported as percentage of CFUs obtained for each condition, compared to infected untreated cells. Data from three repeated experiments are presented as bar plot reporting the mean ± SD. Data regarding are reported in panels **(B, E)** (*E. coli*), **(C, F)** (*S. agalactiae*), and **(D, G)** (*C. albicans*). CFS, cell-free supernatant; MOI, multiplicity of infection; LcM247, Lactobacillus crispatus M247; CFU, colony-forming unit. Statistical signifcance was indicated using asterisks, with the following thresholds: p < 0.05 (*), p < 0.01 (**) and p < 0.0001 (****).

Finally, an *in vitro* infection model was set up based on HeLa to mimic vaginal dysbiosis and demonstrate the antimicrobial activity of LcM247 and its CFS. HeLa cells were infected with *E. coli*, *S. agalactiae* and *C. albicans* at a MOI of 100:1 for bacterial species and 10:1 for the yeast. Two hours later, infected cells were treated with LcM247 (MOI = 500:1), CFS and antibiotics or antifungals at MICs or in combination (LcM247-CFS/gentamicin or LcM247-CFS/fluconazole). CFUs were performed 4 and 24 hours later (**Figure 5A**).

The graph in **Figure 5B** shows that 4 hours after treatment, LcM247 had the worst antibacterial activity against *E. coli*, different from the other treatments, which showed a significant reduction in the bacterial load (**Figure 5B**). Interestingly, CFS was confirmed to be the most effective treatment, also in combination with antibiotics, showing a reduction of ~3 log in *E. coli* CFUs (*p* < 0.0001). In contrast, gentamicin alone showed moderate bactericidal activity, resulting in a lower efficacy than the co-administration of gentamicin and LcM247. Twenty-four hours after infection, gentamicin showed a strong antibacterial effect, reducing the *E. coli* load by ~7 log compared to the untreated sample (*p* < 0.0001) (**Figure 5E**). LcM247 did not eradicate *E. coli*, although co-administration with the antibiotic reduced CFUs by ~5 log (*p* < 0.0001) compared to control. Remarkably, CFS and CFS–gentamicin exhibited complete bacterial elimination (100% reduction compared to the control, *p* < 0.0001).

As depicted in **Figure 5C**, 4 hours post-treatment, LcM247 showed no antibacterial activity on *S. agalactiae*-infected cells, exhibiting a bacterial replication comparable to that of the infected and untreated cells. Interesting, CFS confirmed a significant reduction of ~3 log in the burden of *S. agalactiae*, equivalent to the antibiotic administration alone and in combination with *Lactobacillus* or the supernatant. Notably, even after 24 hours of incubation, LcM247 failed to completely eradicate the bacterial load, although it started to exhibit measurable antimicrobial activity relative to the untreated control, reducing CFUs by approximately 3 log (*p* < 0.0001). The co-administration of gentamicin and CFS emerged as the most effective treatment, exhibiting a complete bactericidal activity (100% of reduction, *p* < 0.0001) (**Figure 5F**). The other treatments, including CFS alone, gentamicin alone and the combination of gentamicin with LcM247, showed a limited antibacterial efficacy, resulting in a minimal reduction in microbial load compared to the 4-hour incubation period (~6 log of reduction compared to the control, *p* < 0.0001).

In the *C. albicans* infection model, only CFS and CFS in association with fluconazole showed a slight reduction of microbial burden following the 4-hour treatment of infected cells (**Figure 5D**). Similarly, 24 hours post-incubation, CFS and CFS–fluconazole administration maintained a significant antimicrobial activity, reducing yeast growth by ~1 log compared to the control (*p* < 0.0001) (**Figure 5G**). In contrast, fluconazole alone or in combination with LcM247 seemed to only have a fungistatic effect (total reduction of ~1 log compared to the control, *p* = 0.0019 and *p* = 0.0020, respectively). Lastly, the LcM247 treatment alone was not able to exert an antimicrobial effect on *C. albicans* (*p* = 0.048).

**Figure 6:**
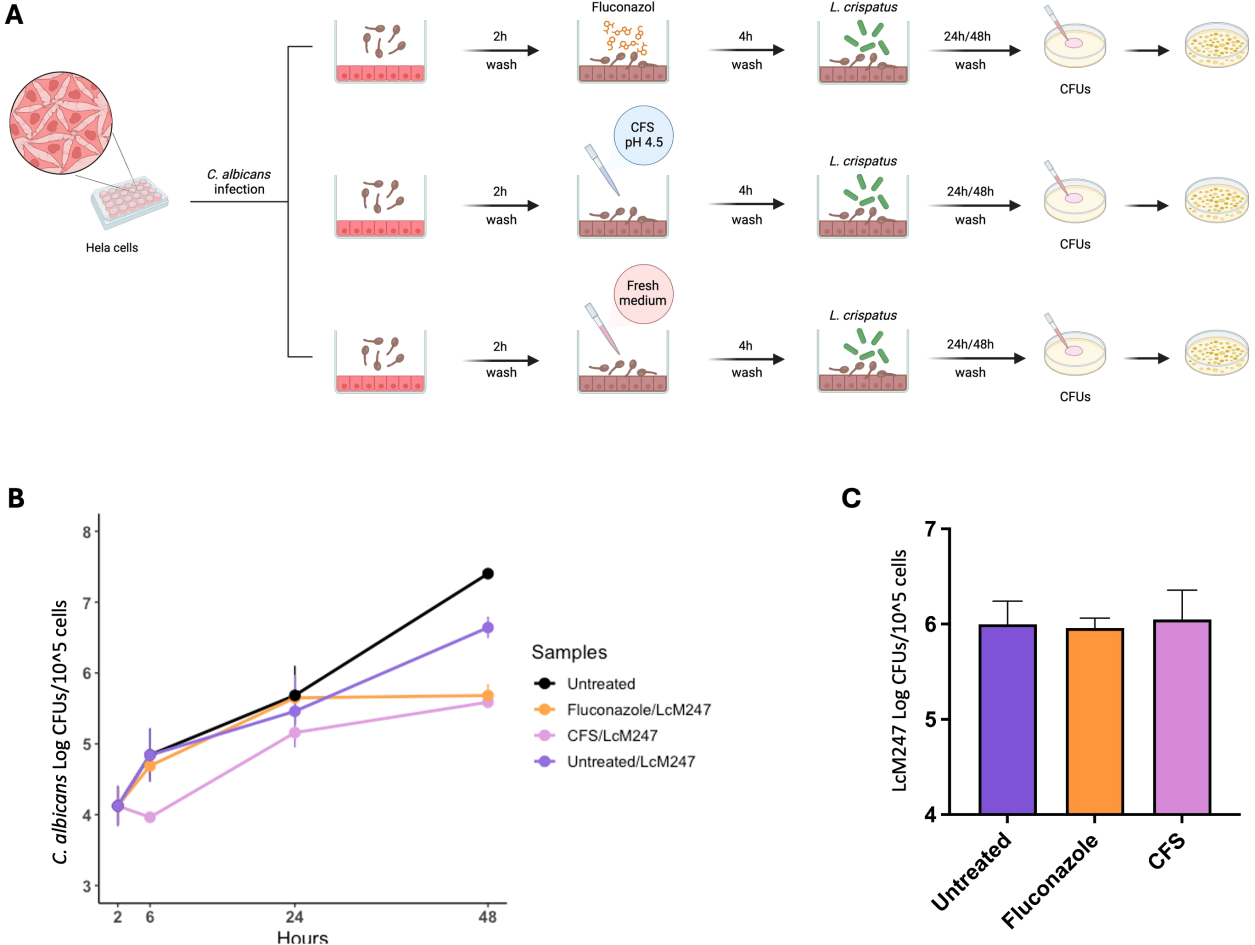
The administration of antimicrobial agents or CFS does not enhance LcM247 engraftment on cells. Schematic representation of the experimental setup. HeLa cells were infected with *Candida albicans* (MOI 10:1) for 2 hours, followed by washes and treatments with fluconazole (at MIC), CFS (CFS v/v 1:1 in culture medium) or fresh medium for 4 hours. After treatment, LcM247 (MOI 500:1) was added to assess its potential for antifungal activity and its engraftment on epithelial cells. CFU counts were assessed after 4, 24 and 48 hours post-infection **(A)**. Line plot showing Log CFUs/10^5^ cells generated in three repeated experiments **(B)**. LcM247 persistence on cells after 48 hours is presented as bar plot reporting the mean ± SD **(C)**. CFS, cell-free supernatant; LcM247, *Lactobacillus crispatus* M247; MOI, multiplicity of infection; CFU, colony-forming unit.

To investigate whether treatment with LcM247 CFS was able to enhance LcM247 engraftment on epithelial cells following an infection, we set up an additional experiment using *C. albicans* that, based on our previous experimental findings, may require longer treatment to be eradicated. HeLa cells were infected with *C. albicans* for two hours, followed by administration of fluconazole, LcM247 CFS (at pH 4.5) or fresh medium. Four hours later, LcM247 was added to each condition. CFUs were assessed at 4, 24 and 48 hours post-infection to assess both antifungal activity and the LcM247 burden (**Figure 6A**).

As depicted in **Figure 6B**, 4 hours post-infection, CFS treatment significantly reduced *C. albicans* load in comparison with fluconazole treatment or untreated control. This trend was maintained by 24 hours post-infection, although the co-activity of antimycotic drug or CFS was supported by LcM247.

Interestingly, 48 hours post-infection, CFS/LcM247 and fluconazole/LcM247 combinations showed a comparable and superior ability to contain *C. albicans* burden compared to untreated cells or cells treated with LcM247 alone.

The data collected suggest that the administration of CFS and antimycotic has a similar efficacy when administered in combination with the live probiotic strains. Of note, LcM247 engraftment appeared independent from antimicrobial pre-treatment (**Figure 6C**), suggesting that *Lactobacillus*’s colonization ability on eukaryotic cells is relatively unaffected by prior exposure to drugs or other treatments.

### CFS reduces microbial burden in infected *G. mellonella*

To corroborate our findings obtained in a monocellular *in vitro* infection model, we assayed CFS antimicrobial activity in a complex but highly reproducible infection model in *G. mellonella*. *G. mellonella* larvae are widely used as a surrogate model of several infectious diseases due to the ease of use and the presence of an innate immune system (Ligeza-Zuber, 2012; Kohler, 2015). LcM247 and CFS cytotoxicity in *G. mellonella* were assessed, showing a good biocompatibility in this model (**Supplementary Figure S3**).

**Figure 7:**
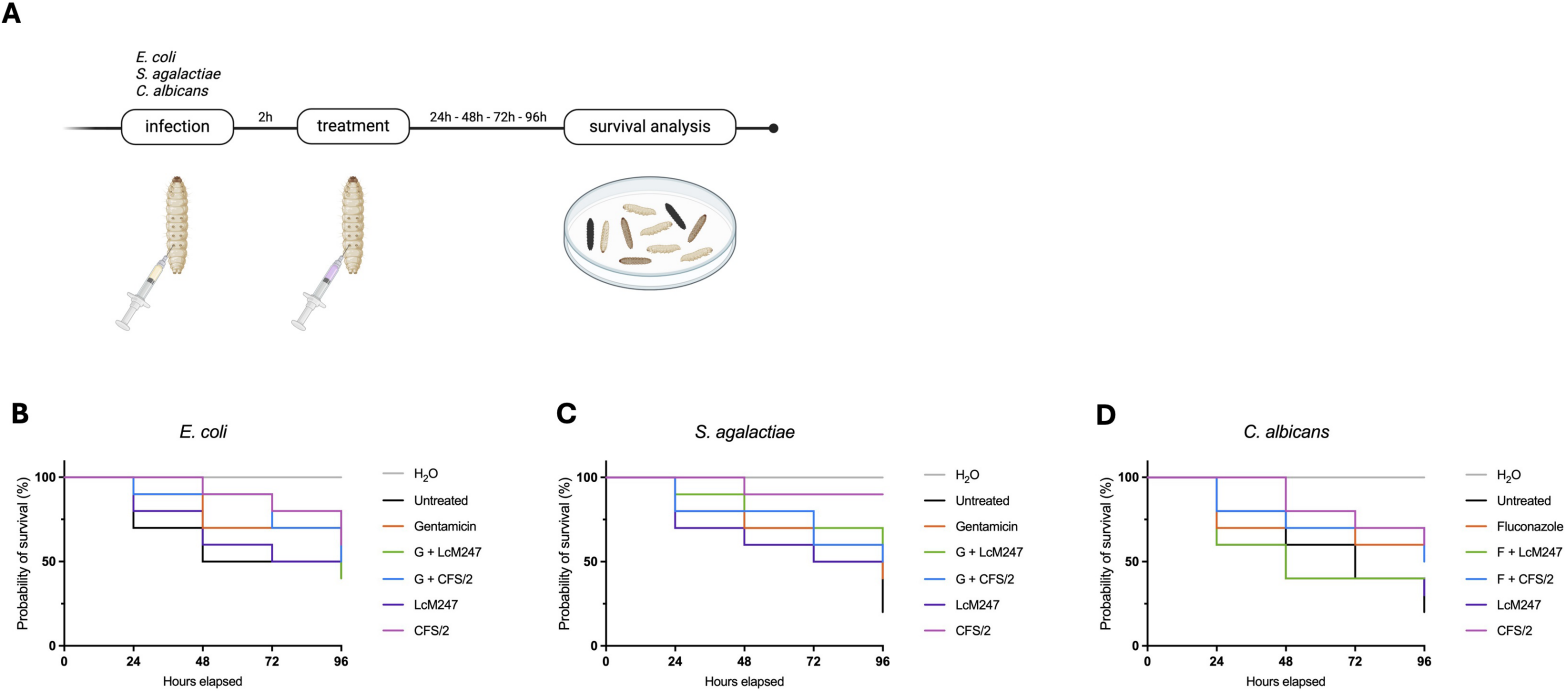
LcM247 CFS prolongs the survival of infected *Galleria mellonella* larvae. Schematic representation depicts the experimental *in vivo* model used to assay antimicrobial activity of LcM247, CFS/2, gentamicin/fluconazole and co-administration of CFS/2 or LcM247 with drugs on infected *G. mellonella* larvae. Ten microliters of infected solution was injected into the last left proleg. Two hours post-infection, 10-µL treatments were inoculated into the last right proleg. For the control groups, 10 µL of sterile H_2_O was inoculated. The number of dead *G. mellonella* was recorded every 24 hours for 4 days **(A)**. Survival curve of *G. mellonella* infected with *Escherichia coli* **(B)**, *Streptococcus agalactiae* **(C)** and *Candida albicans* **(D)** and treated as previously described. LcM247, *Lactobacillus crispatus* M247; CFS, cell-free supernatant.

*G. mellonella* was infected with 10 µL of *E. coli* (5 × 10^5^ CFU/mL), *S. agalactiae* (2 × 10^8^ CFU/mL) and *C. albicans* (5 × 10^7^ CFU/mL). Two hours post-infection, LcM247, CFS/2, gentamicin/fluconazole or their combinations were injected, and larvae were incubated at 37°C for 96 hours (**Figure 7A**). Infected *G. mellonella* were monitored daily, and live ones were counted.

As shown in **Figure 7B**, untreated *G. mellonella* infected with *E. coli* had a survival rate of 40% at 96 hours. The same survival rate was observed in infected larvae co-treated with the combination of gentamicin and LcM247. A slightly higher survival rate (50%) was reached when infected larvae were treated with LcM247 alone, gentamicin alone and gentamicin co-administered with CFS/2. Interestingly, CFS/2 treatment demonstrated the strongest antimicrobial effect in the *in vivo* model, with a minimal mortality of *G. mellonella* larvae observed until the last day of infection (mortality of 10% at 48 hours, 10% at 72 hours and 20% at 96 hours).

The most striking results were observed for *S. agalactiae* infection, which was in line with what was previously observed in the *in vitro* infection model (**Figure 7C**). Indeed, the untreated group of *G. mellonella* larvae had a survival rate of 20%, while antibiotic administration increased survival to 40%. Gentamicin in combination with CFS/2 or LcM247 increased the survival rate up to 50% or 40%, respectively. Of note, CFS/2 administration accounted for 90% larval survival, indicating a high activity against *S. agalactiae*.

Finally, *C. albicans* infection had a survival rate of approximately 20% in infected larvae (**Figure 7D**). When infected larvae were treated with LcM247 or a co-administration of fluconazole and LcM247, the survival rate was enhanced to 30% or 40%, respectively. The co-administration of fluconazole and CFS/2 reduced mortality by 50%, while fluconazole and CFS/2 alone were able to decrease mortality up to 40%.

These findings suggested that CFS has a promising antimicrobial activity in *in vivo* models, even when not co-administered with antimicrobial compounds.

## Discussion

The vaginal microbiome is normally dominated by *Lactobacillus* species, showing low diversity but strong dynamics in changing its composition that can be influenced by various exogenous and endogenous factors (Abou Chacra et al., 2021). Unlike the gut microbiota, the increase in diversity appears associated with a dysbiotic status, such as BV or VVC (Farr et al., 2021; Sun et al., 2023). The clinical aspect of these conditions ranges from an asymptomatic finding in most cases to a clinically symptomatic entity that has a major impact on a woman’s lifestyle (Kroon et al., 2018). At the clinically severe end of the BV spectrum, antibiotic treatment (either systemic or localized) is associated with a 30% recurrence rate within 3 months of initial treatment and up to a 50%–70% recurrence rate within 1 year (Bradshaw et al., 2006). Therapeutic options are very limited in the subpopulation of women who have persistent or recurrent BV despite multiple attempts at antibiotic treatment (Muzny and Kardas, 2020; Muzny et al., 2022). Importantly, the probiotic treatment of symptomatic patients with oral and/or vaginal administration of *Lactobacillus* strains has produced mixed results (Bohbot et al., 2018), suggesting that a complete change of the vaginal microbiome may be required for an effective cure at the clinically severe end of the BV or VVC spectrum. Indeed, the same with fecal microbiota transplantation (FMT), in which feces from healthy donors are introduced into recipients’ intestines to replace their disease-associated microbiome, vaginal microbiota transplantation (VMT) from healthy female donors has recently been proposed as a therapeutic alternative for patients suffering from symptomatic, intractable and recurrent bacterial vaginosis (Lev-Sagie et al., 2019; Yockey et al., 2022).

We confirmed that the antimicrobial activity of LcM247 showed an early bacteriostatic effect during the first 4 hours of incubation and no significant impact on *C. albicans*, while it increased their bactericidal activity during the prolonged incubation of 24 hours, eliminating *E. coli*, *K. pneumoniae*, *S. aureus* and *S. agalactiae* bacteria, pointing out that the growth state of lactobacilli interfere with the adhesion of pathogens. To restore a homeostatic bacterial community, lactobacilli need to properly colonize the mucosa; failure in some treatments may be due to low or insufficient bacterial load due to inaccurate administration or conditions not suitable for proper *Lactobacillus* colonization (or temporary residence, allowing the recovery of native lactobacilli).

Meanwhile, the antimicrobial properties of CFS showed, after 4 hours, a complete inhibition of *E. coli*, *K. pneumoniae* and *S. agalactiae* and a significant reduction of *S. aureus*, *E. faecalis* and *C. albicans*. Interestingly, after 24 hours of incubation, no colonies were detected for all bacterial strains.

The CFS antimicrobial efficacy is closely tied to the pH of CFS, which exhibited an optimal activity at an acidic pH of 4.5, which significantly decreases when the pH was adjusted to neutral (7) or alkaline (9) levels. While additional molecular factors may contribute to the observed antimicrobial effect, proteomic analyses indicated a limited involvement of bacteriocins, with structural components such as S-layer proteins potentially playing a more prominent role under our experimental conditions.

Recent research suggests that *Lactobacillus*-based probiotics may help to restore healthy vaginal microbiota by counteracting potential pathogens (Borges et al., 2014). However, there has been growing interest over the past decade in non-living microorganisms, such as microbial extract or cell-free supernatants, which have shown therapeutic potential and beneficial effects (Pique et al., 2019). The lower diversity of vaginal microbiota compared to gut microbiota and the significant dominance of *Lactobacillus* prompted us to use mono-bacterial probiotics or mono-species probiotics, although recent results obtained via VMT suggest that bacteria together with bacterial products are able to restore a healthy microbial community (Lev-Sagie et al., 2019). This evidence highlights the necessity for further elucidation of the role of probiotics in the treatment of BV/VVC, with a particular focus on determining the most appropriate bacterial strains or products, the optimal timing of administration and the most effective therapeutic strategies. In other words, administrations of probiotics, which need to significantly grow and produce some molecules, may be insufficient to restore vaginal community and to cure the disease. Live bacteria can be administered either topically or orally, often requiring a long time for bacteria to reach the target site, establish themselves and begin to act. This is due to the need for live bacteria to colonize the site, a process that can be slow and subject to host and environmental factors. Indeed, studies have shown that the colonization and activity of probiotics can be inconsistent and slow to manifest (Reid et al., 2011; Borges et al., 2014). In contrast, CFS can be administered directly to the site of the infection, and thanks to its high load of bioactive molecules, including bacteriocins and its acidity, it can exert an antimicrobial effect immediately after application. In addition, non-living bacterial treatments circumvent the safety concerns associated with the use of live microorganisms, particularly in vulnerable populations such as those with compromised immune systems or during pregnancy, such as transmissible antibiotic resistance genes, systemic infections due to the bacterial translocation in human sterile sites, metabolomic disturbances and allergic responses. This makes them a potentially safer and more effective alternative to restoring healthy microbiota and inhibiting pathogenic organisms (Kothari et al., 2019).

In this context, we propose a sequential scheme to assay *Lactobacillus* activity against a series of indicator strains that represent some of the most etiological agents of BV or VVC. As a model of *Lactobacillus*, we assessed properties of LcM247, one of the most used *Lactobacillus* species to treat a wide spectrum of disorders including the dysregulation of the vaginal flora (Cesena et al., 2001; Zhang et al., 2018; Di Pierro et al., 2023b; Di Pierro et al., 2023a).

The use of CFS from lactobacilli for treating vaginal infections offers several advantages over traditional antibiotics or antifungals. Firstly, CFS contains multiple antimicrobial compounds that can target a broad spectrum of pathogens without promoting resistance (O’Hanlon et al., 2013; Fagan and Fairweather, 2014; Kovachev, 2018; Abdul-Rahim et al., 2021; Saha and Saroj, 2022; Assandri et al., 2023; Decout et al., 2024), a significant issue with traditional antibiotics and antifungals. Indeed, the overuse of antibiotics has led to an increase in multidrug-resistant organisms. Secondly, CFS can help to restore and maintain the natural vaginal microbiota, preventing recurring infections, while antibiotics disrupt the delicate balance of the vaginal microbiome, killing also the beneficial bacteria like lactobacilli (Ahrens et al., 2020; Ma et al., 2022).

The proteomic profile of CFS highlights the absence of a significant amount of secreted bacteriocins and, in parallel, the presence of S-layer proteins and metabolic enzymes, which thereby emerge as the most likely candidates for the observed antimicrobial activity of LcM247 CFS. The S-layer proteins form a protective layer that warrants bacterial structural integrity and mediates interactions with the environment (Hynonen and Palva, 2013; Angelescu et al., 2024). The high abundance of S-layer in the CFS results from the non-covalent interaction with bacterial cell surface carbohydrates, and this labile attachment to the bacterial cells may be very important to exert its activity in the surrounding environment (Hynonen and Palva, 2013; Fagan and Fairweather, 2014).

S-layers are made of relatively small protein subunits with a crystalline and regular structure that can quickly and efficiently refold into their active configuration and retain their functional properties, including bactericidal activity (Darbandi et al., 2022; Sugrue et al., 2024). Indeed, S-layer proteins are resistant to heat inactivation at 95°C in the free form (Sara and Sleytr, 2000; Frece et al., 2005); this thermal resistance ensures that the antimicrobial compounds produced by lactobacilli remain effective even after exposure to elevated temperatures. Moreover, S-layer proteins present a high content of acidic and hydrophobic amino acids (isoelectric point in acidic pH range) and lysine as a basic amino acid and a low content of arginine, histidine, methionine and cysteine (Sara and Sleytr, 1996). Of note, S-layer proteins are small proteins that contain few tryptophan residues that are the main target of proteinase K. Hence, the findings that CFS maintains its antimicrobial activity despite heat inactivation and proteinase K treatment are consistent with the possibility that the *Lactobacillus* S-layer is implicated in this activity.

The biological functions of *Lactobacillus* S-layer proteins are still not fully elucidated, even though many of these proteins appear to mediate bacterial adherence to host cells or interaction with host immune mediators or show protective or enzymatic functions, at least in non-pathogenic bacteria (Hynonen and Palva, 2013; Klotz et al., 2020; Assandri et al., 2023; Decout et al., 2024). In this context, only one study indicated the S-layer protein of *Lactobacillus acidophilus* to have murein hydrolase activity against *Salmonella enterica* (Prado Acosta et al., 2008). Intriguingly, an attempt to exploit these antimicrobial properties was made by Rao and colleagues, who linked the S-layer protein isolated from *Lactobacillus buchneri* to silver nanoparticles and assayed the antibacterial activity of this preparation against *S. enterica* and *S. aureus*. The authors observed a reduction of the MIC of their compound in comparison with silver nanoparticles alone, a higher antibiofilm activity, an increased cell membrane permeability and stronger inhibition of respiratory-chain dehydrogenase activity in treated bacteria (Rao et al., 2022). Unfortunately, the activity of the S-layer alone was not evaluated in the proper environment. It will also be important to assess the antimicrobial activity of S-layers from different *Lactobacillus* species or strains, considering that S-layer proteins show an overall low amino acid sequence similarity. These heterogeneities in sequence among *Lactobacillus* S-layer proteins may explain the differential activity of CFS originating from different species, such as the CFS from LcM247 and *L. iners* CFS or other *Lactobacillus* CFSs.

While components like S-layer proteins may contribute to antimicrobial effects, the impact of lactic acid should be critically considered (Sablon et al., 2000; Zangl et al., 2020). Indeed, lactic acid exerts antimicrobial activity, primarily in its protonated form at low pH, enabling it to permeabilize microbial membranes and, in its dissociated form, to interfere with the intracellular processes, inhibiting microbial growth or causing cell death (Diez-Gonzalez and Russell, 1997; Alakomi et al., 2000; Sablon et al., 2000). CFS’s pH-dependent activity, along with its heat and protease insensitivity, may be consistent with lactic acid activity; also, the reduced efficacy of the CFS against *C. albicans* may be explained by its relatively high lactic acid tolerance (Zangl et al., 2020). However, the protonated form predominates when pH < pKa (commonly recognized as approximately 3.8), and previous investigations have explored lactic acid antimicrobial activity only in conditions around this point (pH 3.6 and pH 4.0) and mainly on gram-negative bacteria (Alakomi et al., 2000). Finally, the existence of distinct, individual effects of an initial pH and initial undissociated lactic acid concentration on the bacterial inactivation suggests that lactic acid antimicrobial activity may be characterized by a certain variability (Janssen et al., 2007), an effect that we did not observe in our setting. Although we acknowledge that this may be a limitation of the current study, our findings open to further investigations that may address the complex interaction among pH, lactic acid, bacteriocins and S-layer proteins in their overall synergistic antimicrobial activity in the multifaceted field of lactic acid bacteria (Saha and Saroj, 2022).

LcM247 and particularly its CFS have been shown to contribute to the eradication of common pathobionts, suggesting that commercially available probiotics based on this strain may effectively restore a healthy vaginal microbiome. We clearly show that actively replicating LcM247 is less efficient than its CFS, so the oral administration of LcM247 may result in the failure of the treatment. Finally, the use of CFS may result in an upswing of the host *L. crispatus* strains, which conversely may show a minor tolerance to antibiotic treatment (Costantini et al., 2021). However, our findings together with phylogenetic information regarding *L. crispatus* strains suggest that the identification of CST-I alone may be not sufficient to describe a healthy vaginal community, prompting for a fine and deep characterization of more strains. As previously demonstrated, significant differences emerged when investigating *L. crispatus* strains isolated from human hosts or other hosts, as well as isolated from diverse host biological environments, intrinsically displaying different growth patterns and impacting their colonization and persistence (Pan et al., 2019; Hulyalkar et al., 2021). A better understanding of these strains may clarify their role in preserving and regulating vaginal homeostasis during the fertile age.

## Data Availability

The datasets presented in this study can be found in online repositories. The names of the repository/repositories and accession number(s) can be found in the article/**Supplementary Material**.

## Supplementary material

Supplementary Figure 1 CFS pH measurement corresponding to serial dilutions and alkalinization. LcM247 CFS was serially diluted at a ratio of 1:1 v/v in MRS broth and pH was measured for each obtained condition. Additionally, CFS was alkalinized by adding different volumes of 1M NaOH until four different conditions were achieved: unaltered CFS (pH 4.5), CFS at pH 5.5, CFS at pH 7, and CFS at pH 9.

Supplementary Figure 2 LcM247 CFS and *L. iners* CFS does not have a reciprocal inhibition. Preserving a healthy vaginal microbiota depends on the *Lactobacillus* species that dominate the vaginal microenvironment. The protective role of LcM247 was assessed against *L. iners* (typically identifying CST-III) associated with microbiota prone to develop disease. We investigate the interaction between the supernatant of *L. crispatus* M247 (LcM247) and *L. iners* cells, and the interaction between the supernatant of *L. iners* and LcM247. We performed an antimicrobial assay mixing serial dilutions of CFS and Lactobacillus v/v 1:1 and incubating for 24 hours at 37°C. Untreated lactobacilli, incubating in MRS medium v/v 1:1 was used as positive control. Finally, colony forming units (CFUs) were assessed and two bar plots showing Log CFUs/ml of *L. iners* after incubation with LcM247 CFS **(A)** and Log CFUs/ml of LcM247 incubating with *L. iners* CFS **(B)** were generated by measures of three repeated experiments.

Supplementary Figure 3
*In vivo* toxicity. Toxicity survival curve of gentamicin, fluconazole, LcM247 and CFS/2 and their combinations on *G. mellonella* larvae monitored every 24 hours till 96 hours. *G. mellonella* larvae were inoculated with 10 µl of 10 µg/ml gentamicin/fluconazole, 5 x 10^7 LcM247, CFS/2 (1:1 v/v) and with the combination of these compounds.

Supplementary Table 1 Bacteriocins detected in *Lactobacillus crispatus* M247 from BAGEL 4. The document includes a table listing the Areas of Interest (AOI) for each bacteriocin gene detected from BAGEL 4 in the LcM247 genome, specifying the start and end positions. Additionally, the corresponding nucleotide and protein sequences for every identified bacteriocin are included.

Supplementary Table 2 Peptides detected by proteomic analysis.

Supplementary Table 3 Comprehensive Proteomic Analysis of the Cell-Free Supernatant (CFS) from LcM247 founded with *Lactobacillus crispatus* database.

Supplementary Table 4 Comprehensive Proteomic Analysis of the Cell-Free Supernatant (CFS) from LcM247 founded with *Lactobacillus* genus database.
