# Supplementary figures and images for: The activity of cell-free supernatant of Lactobacillus crispatus M247: a promising treatment against vaginal infections
Source: Front Cell Infect Microbiol. 2025 Jun 11;15:1586442. doi: 10.3389/fcimb.2025.1586442 (PMC12187682; doi:10.3389/fcimb.2025.1586442)

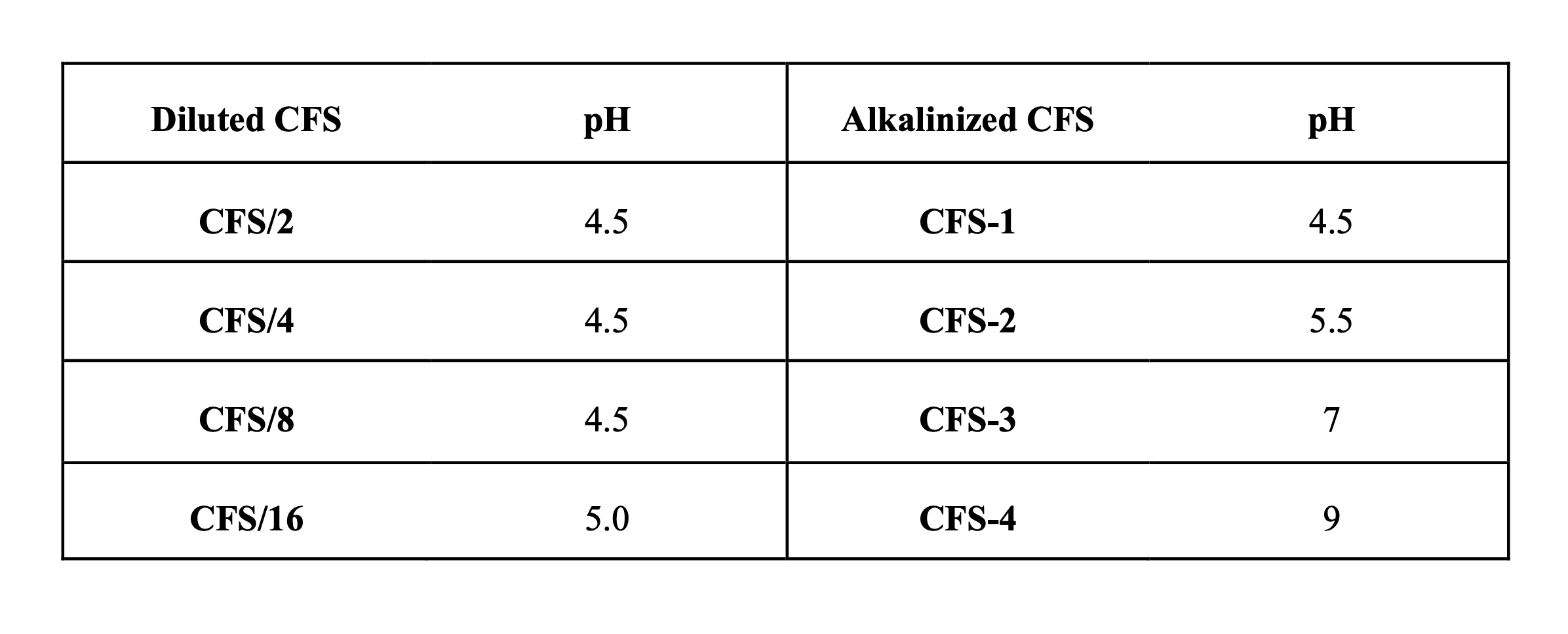

Supplement: Supplementary Figure 1 — CFS pH measurement corresponding to serial dilutions and alkalinization. LcM247 CFS was serially diluted at a ratio of 1:1 v/v in MRS broth and pH was measured for each obtained condition. Additionally, CFS was alkalinized by adding different volumes of 1M NaOH until four different conditions were achieved: unaltered CFS (pH 4.5), CFS at pH 5.5, CFS at pH 7, and CFS at pH 9. [file Image1.png]

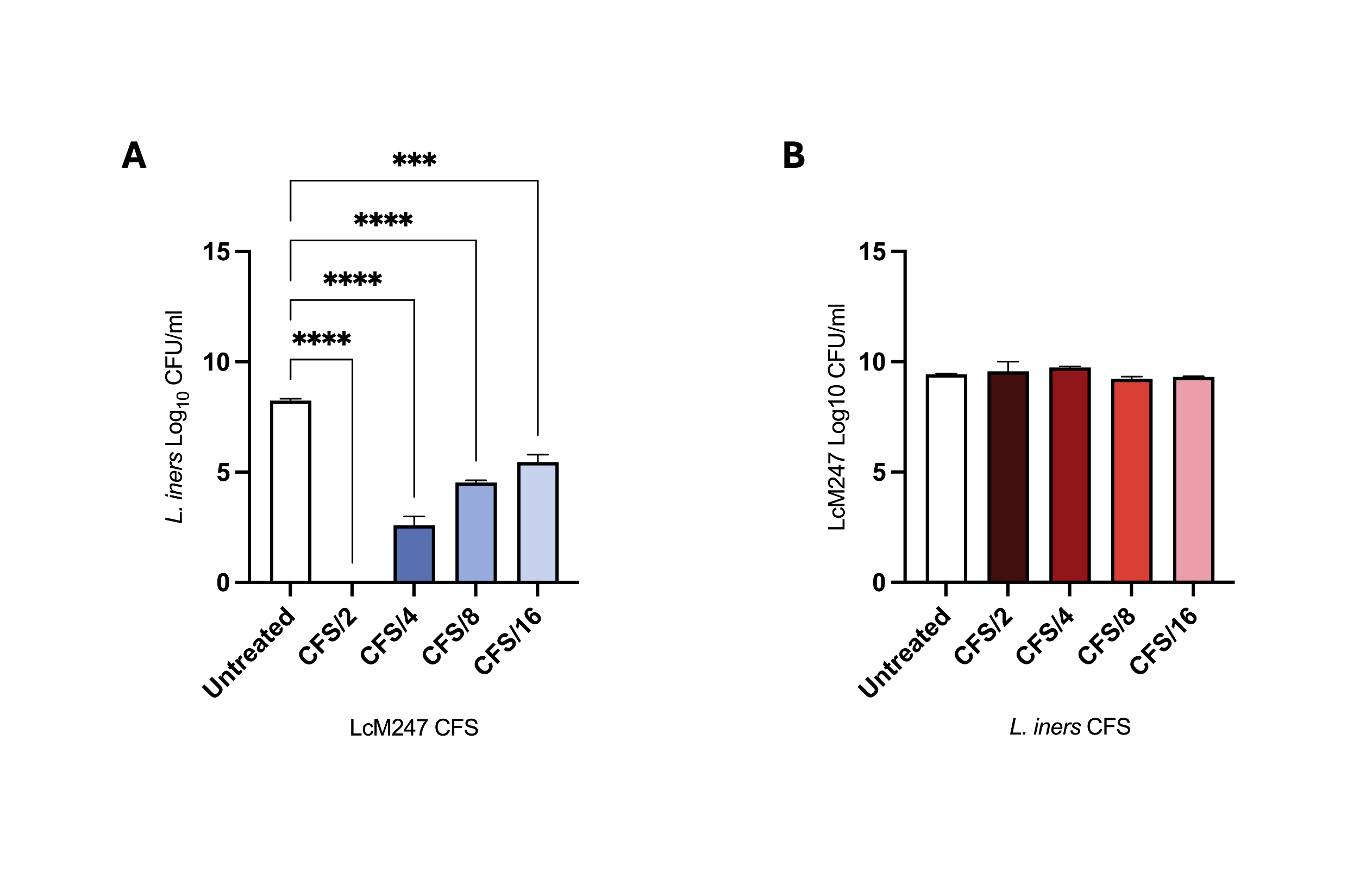

Supplement: Supplementary Figure 2 — LcM247 CFS and L. iners CFS does not have a reciprocal inhibition. Preserving a healthy vaginal microbiota depends on the Lactobacillus species that dominate the vaginal microenvironment. The protective role of LcM247 was assessed against L. iners (typically identifying CST-III) associated with microbiota prone to develop disease. We investigate the interaction between the supernatant of L. crispatus M247 (LcM247) and L. iners cells, and the interaction between the supernatant of L. iners and LcM247. We performed an antimicrobial assay mixing serial dilutions of CFS and Lactobacillus v/v 1:1 and incubating for 24 hours at 37°C. Untreated lactobacilli, incubating in MRS medium v/v 1:1 was used as positive control. Finally, colony forming units (CFUs) were assessed and two bar plots showing Log CFUs/ml of L. iners after incubation with LcM247 CFS (A) and Log CFUs/ml of LcM247 incubating with L. iners CFS (B) were generated by measures of three repeated experiments. [file Image2.png]

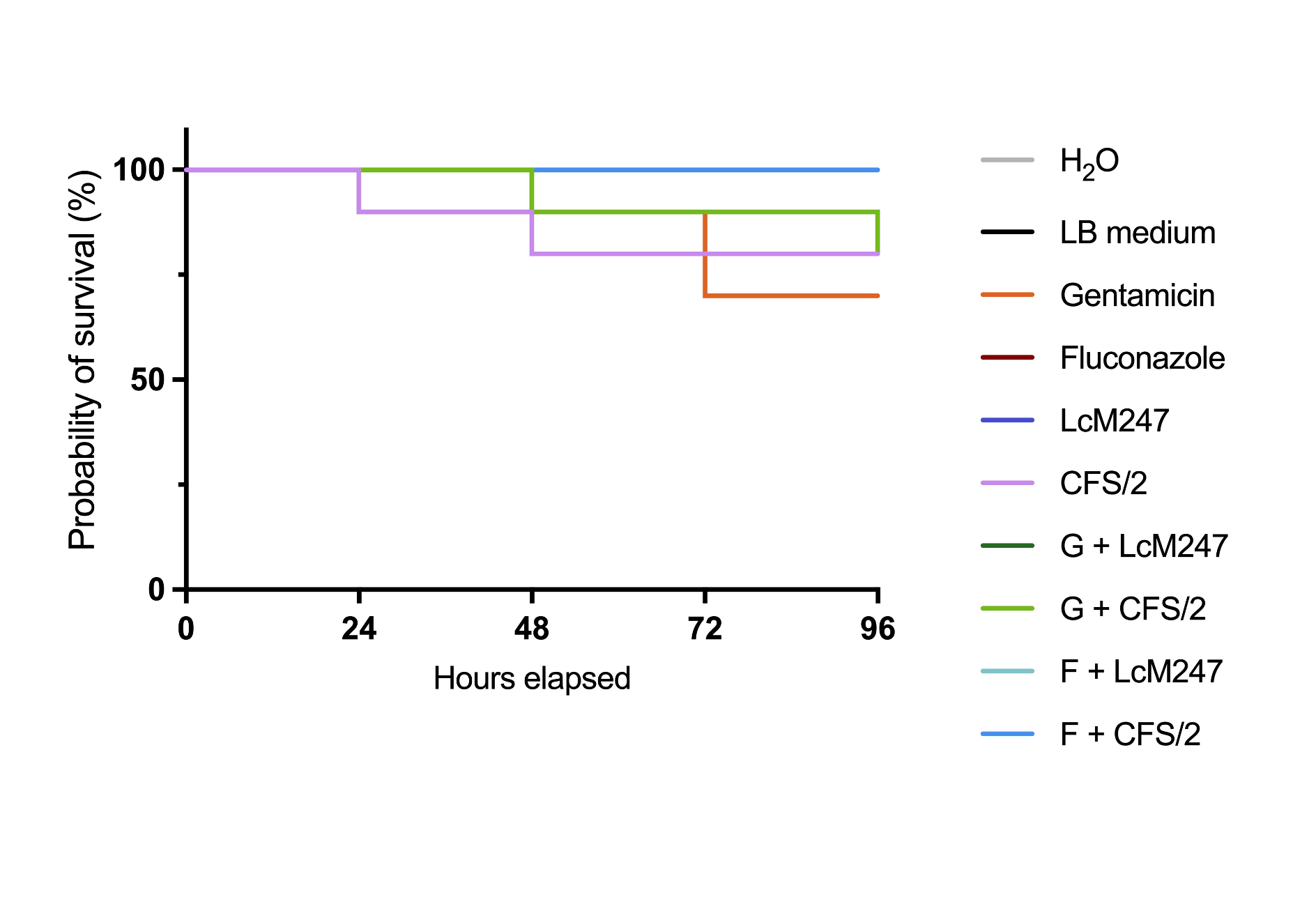

Supplement: Supplementary Figure 3 — In vivo toxicity. Toxicity survival curve of gentamicin, fluconazole, LcM247 and CFS/2 and their combinations on G. mellonella larvae monitored every 24 hours till 96 hours. G. mellonella larvae were inoculated with 10 µl of 10 µg/ml gentamicin/fluconazole, 5 x 10^7 LcM247, CFS/2 (1:1 v/v) and with the combination of these compounds. [file Image3.png]
