# Supplementary material for: The activity of cell-free supernatant of Lactobacillus crispatus M247: a promising treatment against vaginal infections
Source: Front Cell Infect Microbiol. 2025 Jun 11;15:1586442. doi: 10.3389/fcimb.2025.1586442 (PMC12187682; doi:10.3389/fcimb.2025.1586442)
Supplement: Supplementary Table 1 — Bacteriocins detected in Lactobacillus crispatus M247 from BAGEL 4. The document includes a table listing the Areas of Interest (AOI) for each bacteriocin gene detected from BAGEL 4 in the LcM247 genome, specifying the start and end positions. Additionally, the corresponding nucleotide and protein sequences for every identified bacteriocin are included. [file Table1.docx]

| AOI | Start | End | Class |
| --- | --- | --- | --- |
| 30.29.AOI_01 | 0 | 15984 | 70.3;Helveticin-J |
| 107.31.AOI_01 | 1 | 4359 | 64.3;Enterolysin_A |
| 91.47.AOI_01 | 0 | 5928 | 163.2;Penocin_A |
| 7.50.AOI_01 | 20144 | 34952 | 6.3;Bacteriocin_helveticin_J |
| 58.5.AOI_01 | 1 | 11005 | 163.2;Penocin_A |
| 8.66.AOI_01 | 12722 | 32962 | 210.2;SakT_alpha |
| 100.98.AOI_01 | 0 | 4890 | 64.3;Enterolysin_A |

**70.3;Helveticin-J**

**Gene sequence**

ATGTTAGATGCAACAAATATTAGAATTTTAGATAGATTCGATATGAATACAGGTTACAGAGCTGTAGTTCAAAAAGGTAATGTAGGATCAAAGTATGTTTATGGTTTACAGTTAAGAAATAACCAGACAGAAACACATGTATTGCGTGGCTTCCGTGGCAACGTGACAACCCCTGTGCTTACTTTAGTAGGTGCAGCAGCTGGACACACCCAAACTTGGGAATATTCAGGTAGATCTGGTAAATGGTTCGTTGGTACTAAAAACAAGAATAAATGGGCTTCTCAAATTGCTCGAGCAGATATTAGATACAAATCATATGCTTCATCAAATACTGAATTCCCACGTTTAGCATACTTGAACCGCGCAGGTAATCCTGAAGATCAATGCTCTGGTGATGAAATGGAACGCGCTGAAGTAGCAGTTTCACCAGATTATAGTATGCTTTTAATTGCTACTATTGAGAATAATGGAACAGGCCACTTTTCAATTTATGACCTAAATGTCATTAATAATGCATTAGATGAGGCTGGAAATAATGGTTTCGTAAATATGGGAAATTATCAATGTGAAACTAGTTTTACAGTATATGGTTTGTATGGATCTGTTTTAAACTCTGTACAAGGTTATGATCTTGATAATTCTGGTAATATTTATATTACTAGTCAAAAATCACCAAGTTTAAGTAATGGTTCATGGTCATACTATCACAAGGAAATTGTAAAAATTCCGTTTTATGCACGTAATGATCAATCTCAATGGGAAAATGTAAACTTAAGTGCATTTGGTGGATTAGATATTTCAGGTGAACATAGTGAAGTTGAAAGTATCCAAATTATTGATGAAAATCACGGTTACTTAACTGTTGCATACCATGCTAATGTTAACGGTAAAAATAAAACTGTTTCAAATAAGGTTTATGAATTAGCTTGGGAC

**Protein sequence**

MLDATNIRILDRFDMNTGYRAVVQKGNVGSKYVYGLQLRNNQTETHVLRGFRGNVTTPVLTLVGAAAGHTQTWEYSGRSGKWFVGTKNKNKWASQIARADIRYKSYASSNTEFPRLAYLNRAGNPEDQCSGDEMERAEVAVSPDYSMLLIATIENNGTGHFSIYDLNVINNALDEAGNNGFVNMGNYQCETSFTVYGLYGSVLNSVQGYDLDNSGNIYITSQKSPSLSNGSWSYYHKEIVKIPFYARNDQSQWENVNLSAFGGLDISGEHSEVESIQIIDENHGYLTVAYHANVNGKNKTVSNKVYELAWD

**64.3;Enterolysin_A**

**Gene sequence**

ATGAAAAAAACTGAAAGTAAGTTTGCACTTTTAGCTGCTTTAATTGCTATTTTAGCTTTTGCCTCTATTCCCTTATGGCAAAACAATCTGAATAGTTTACGACCACAAACTCACACGGTTAAGAAAAAGAAGACTGCTAAAAAGAAAAAAGTCGTTCATCTTACTTGGGGTTACCCTTTTAAGAAGCTCTATGAAAAGAAAATCAAATTTAAATCTGGACAAAAGTTTGGTGAAACTGACGTCATTCGTCGAGTCTATCCTAGCAAGAGTTATTTTCATGATGGCTATGACTTCGGATTTAGTGAAGTTGGACATTCTTCCGTATATGCTGTGCATGCAGGAACTGTCCATCGTGTTAAATACGCCCCAGGTTTAGGACTTTATATTTGGATTATTTCCGATGATGGCTATGTTGAAGTTTACCAGGAAGGCTTCCTCAGTATTACTGATATTTATGTTAAAAAAGGTCAAAAAGTCAAGTTGGGACAAAAAATTGGTAAACTGACCGGCTCTCACATTCATTTAGGAGTTACTAAGACAGATAAAGATTACATTGATAAAAAGCATGACAATACGCCATGCAAATACTATTGGAAAGATAATGGCACTTGGTTAAATCCAATGAAAATCATTGAAGATAATCTAAGGGCAGCAGGTAAAGATCCAGTCCAA

**Protein sequence**

MKKTESKFALLAALIAILAFASIPLWQNNLNSLRPQTHTVKKKKTAKKKKVVHLTWGYPFKKLYEKKIKFKSGQKFGETDVIRRVYPSKSYFHDGYDFGFSEVGHSSVYAVHAGTVHRVKYAPGLGLYIWIISDDGYVEVYQEGFLSITDIYVKKGQKVKLGQKIGKLTGSHIHLGVTKTDKDYIDKKHDNTPCKYYWKDNGTWLNPMKIIEDNLRAAGKDPVQ

**163.2;Penocin_A**

**Gene sequence**

ATGAAGAATTTTAAGGTAGTAAATAATATTGAGCTAAATAGAGTTGTTGGTGGTAAAATTATTCGATTAACTCCATATATGTTGTATAATACTAAGACACATAAAACTATCCCAGATTACGGTGCAATTTGGGGTAAAGCGGGTCAAACCGTAGCCAATGGATGGTTACAATATGGACCATGGGGCAGTAGAGGT

**Protein sequence**

MKNFKVVNNIELNRVVGGKIIRLTPYMLYNTKTHKTIPDYGAIWGKAGQTVANGWLQYGPWGSRG

**6.3;Bacteriocin_helveticin_J**

**Gene sequence**

ATGGTTAAAAGTATTACACCTCATTTGGTTTATCACTTGAATGGGATGCACCATGTTGTAGCACAAGTTGGTGTAGTAAATGGTGATCATGTTTTTGCCTTGCAACTTCTTCACAGTGCACATGATGTGTTGGTTTATAGAAAACATGAAGGTTTAACCAAGAACATCGATTATACTGATCCACACTTAGTAATGATGGGCTTTGGCCACACGCAAACCTGGGTACCAGCTAATGACAAGGATGAATATTTCGTAGGTGCTAAACCAAATTCAGGCAACTGGACTACTCAAATTGCACGAGTAAAGTACCCAAGACTTTTACCAGAAAGATATACTTCAAATACACAACTTCCACGTTTGTCACACTTGAATCACGTAACCGACGTTCCTTATGATGGTCATGATCACTTGCACAGAGTAGAAGCTTCAGTTTCACCAAATGGCAAGTATTTCATGATTGCCTCAATTTGGGATGATGGTTCAGGTCACTTTGGTTTGTTTGACTTAAATGAAGTAAACCAAAAGTTGAATGAAAATGGCACTAAGAACACGCCAATCACTGATTTACATTGCTTGAGTGCTTTCCACATCGACAACTTCGATAATCCAAGTGTTGCTCCAGATGAAGAAATGCTACAAATGATTGATTCAGTTCAAGGCTATGCTATTGATGATGACAAGAATATCTATATTTCTAACCAATTGTCACCAAAGATTAACCATGAAACTGGTGAAGTAACTACTTGGTCACGTAAGATTGTTAAGTTCCCATGGGGTGAAACAAACAGTGATAACTGGCAAGTAGCTATGGTTGATGGTATTGATTTACCAGATCGTTACAGTGAAATGGAAAGTATCCATGTTAATGCCGCAAATGATATTTACTTAACTGTGGCTTACCACCAAAAGTACATTAAGGGTGGAGAATATAAGTTAAGAACTTTGGAAAACCAAATTTTCCATATTACTGATTTG

**Protein sequence**

MVKSITPHLVYHLNGMHHVVAQVGVVNGDHVFALQLLHSAHDVLVYRKHEGLTKNIDYTDPHLVMMGFGHTQTWVPANDKDEYFVGAKPNSGNWTTQIARVKYPRLLPERYTSNTQLPRLSHLNHVTDVPYDGHDHLHRVEASVSPNGKYFMIASIWDDGSGHFGLFDLNEVNQKLNENGTKNTPITDLHCLSAFHIDNFDNPSVAPDEEMLQMIDSVQGYAIDDDKNIYISNQLSPKINHETGEVTTWSRKIVKFPWGETNSDNWQVAMVDGIDLPDRYSEMESIHVNAANDIYLTVAYHQKYIKGGEYKLRTLENQIFHITDL

**163.2;Penocin_A**

**Gene sequence**

ATGGTAAAGTTTCAAGAACTAAAAGAAAATCAATTAAGTCAAGTTTTAGGCGGTACACATCACAAGCGCGGAGGCGGAAAGTACCATTACTATGGTAATGGTGTATATTGCAACCGATATTATTGTCATGATAATTTAGCACAAATGTGGGATAGCGTTGGTCGTATTATGTATACTGGATGGCAGAAGGATGGACCATTTGCGCATCCTTTGGTG

**Protein sequence**

MVKFQELKENQLSQVLGGTHHKRGGGKYHYYGNGVYCNRYYCHDNLAQMWDSVGRIMYTGWQKDGPFAHPLV

**64.3;Enterolysin_A**

**Gene sequence**

ATGAAATTTCGTAAATTAATAATTTCTTTGTTAGGTACTGCATTATTAACTTCAAGCGTTGGTTTATCAACCACCACTGCTTCTGCTGATACGCTTGATGACTCACAAAATACAACTGAAGTTCAACCCAAGAACCTCAAGTGGGCTTATCCGTTTAAAGCCAACAAGAAAAATGGTGTTCGTCCAATGTATAATGCACAAACTTTTGGCATAACTAACTATATGCGTTCTACTACACCACCTTCCTACTTTCATGATGGTTGGGATTTTGGTTTTTCAGAAGTTGGGCATTCTAACGTATATGCAATTCATCAAGGTACTGTTAAAAAGGTTGCTTATGGCAACGGCCTTGGCTGGTTCATCTGGGTTATTAGTCCTGATAATTACGTTGAAGTTTACCAAGAAGGGTTTAATAAGAAAAAAGACATTTATGTTAAGACTGGTCAAAAGATCGGTAAGCTAACTGGCTCACACTTACACCTTGGCGTTACTCAAACAAATAAAGATTACATAAACAAATATGGTTTTCCATGTAAGAATTGGAACGTTAACAACGGAACTTGGCTCAATCCAATCGAGGTTATCAAAAGCAACTTAAAGAAA

**Protein sequence**

MKFRKLIISLLGTALLTSSVGLSTTTASADTLDDSQNTTEVQPKNLKWAYPFKANKKNGVRPMYNAQTFGITNYMRSTTPPSYFHDGWDFGFSEVGHSNVYAIHQGTVKKVAYGNGLGWFIWVISPDNYVEVYQEGFNKKKDIYVKTGQKIGKLTGSHLHLGVTQTNKDYINKYGFPCKNWNVNNGTWLNPIEVIKSNLKK

**210.2;SakT_alpha/bacteriocinLS2chaina**

**Gene sequence**

ATGAAAAAATTAATTTTGAATTTGCTTACGATTTTTGCTTGGATTTACCAAATATTTTGTGTGATAGGAATAATTATGTGGATCGTTATGGGAGGTGTTGTGTTATTTGGTATACATAATCCGGATTTTCGTGCTGGCTTTGAAAGTGGTAGGGGTATAAAAGGCGTATCAGTTGATAACTATATCGGTGCAATAGTGGTTGTGTTGCTATCACTGATCATGATGAGCGTTGCAGCCTTTTTAATCTGTCGATATGCCAGATTAATTGTTAAGAATATCAAGCAAGAAGTTTACTTTGCTGATAGTAATTTGAATTTACTCAAGAAATTATTAATTTCAGTTGCTGGATATACCCTTATTTCAATAATAGATTACATTATTTTCATAACTCACCGCACTTGGTTCGCTAAATCCTCGAATAATGTACTATACCCAAGTGGTGTGACAACTGGTTTGCTTTTCCTAGCAGTACTATACGTAGTCTACTTGGTATTCAAGTACGGAATGAAGGTTCAGGAGGATGCAGACTCGATTATT

**Protein sequence**

MKIVIRSFIKGKNMNKFETLTYEELSAVFGGNGGKARRRRKITNCAKAIGMGALKDGLKYGIAGTAFGTPIGTVGGAIFGANVGIISGSVSCVSHL
